# Uncovering the genomic basis of an extraordinary plant invasion

**DOI:** 10.1126/sciadv.abo5115

**Published:** 2022-08-24

**Authors:** Vanessa C. Bieker, Paul Battlay, Bent Petersen, Xin Sun, Jonathan Wilson, Jaelle C. Brealey, François Bretagnolle, Kristin Nurkowski, Chris Lee, Fátima Sánchez Barreiro, Gregory L. Owens, Jacqueline Y. Lee, Fabian L. Kellner, Lotte van Boheeman, Shyam Gopalakrishnan, Myriam Gaudeul, Heinz Mueller-Schaerer, Suzanne Lommen, Gerhard Karrer, Bruno Chauvel, Yan Sun, Bojan Kostantinovic, Love Dalén, Péter Poczai, Loren H. Rieseberg, M. Thomas P. Gilbert, Kathryn A. Hodgins, Michael D. Martin

**Affiliations:** ^1^ Department of Natural History, NTNU University Museum, Norwegian University of Science and Technology (NTNU), Trondheim, Norway.; ^2^ School of Biological Sciences, Monash University, Melbourne, Australia.; ^3^ Center for Evolutionary Hologenomics, GLOBE Institute, University of Copenhagen, Copenhagen, Denmark.; ^4^ Centre of Excellence for Omics-Driven Computational Biodiscovery (COMBio), AIMST University, 08100 Kedah, Malaysia.; ^5^ UMR CNRS/uB 6282 Biogéosciences, Université de Bourgogne-Franche-Comté, Dijon, France.; ^6^ Department of Biology, University of Victoria, Victoria, Canada.; ^7^ Institut de Systématique Evolution Biodiversité (ISYEB), Muséum National d’Histoire Naturelle, CNRS, SU, EPHE, UA, National Herbarium (P), 57 rue Cuvier, CP39, 75005 Paris, France.; ^8^ Department of Biology, University of Fribourg, Fribourg, Switzerland.; ^9^ Institute of Biology, Section Plant Ecology and Phytochemistry, Leiden University, P.O. Box 9505, 2300 RA Leiden, Netherlands.; ^10^ Koppert Biological Systems, Department R&D Macrobiology, Veilingweg 14, 2651 BE Berkel en Rodenrijs, Netherlands.; ^11^ Department of Integrative Biology and Biodiversity Research, University of Natural Resources and Life Sciences Vienna, Vienna, Austria.; ^12^ UMR Agroécologie, Institut Agro, INRAE, Univ. Bourgogne, Univ. Bourgogne-Franche-Comté, F-21000 Dijon, France.; ^13^ College of Resources and Environment, Huazhong Agricultural University, Wuhan, China.; ^14^ Department of Environmental and Plant Protection, Faculty of Agriculture, University of Novi Sad, Novi Sad, Serbia.; ^15^ Centre for Palaeogenetics, Stockholm, Sweden.; ^16^ Department of Bioinformatics and Genetics, Swedish Museum of Natural History, Stockholm, Sweden.; ^17^ Botany Unit, Finnish Museum of Natural History, University of Helsinki, Helsinki, Finland.; ^18^ Faculty of Biological and Environmental Sciences, University of Helsinki, Helsinki, Finland.; ^19^ Institute of Advanced Studies Kőszeg (iASK), Kőszeg, Hungary.; ^20^ Department of Botany and Biodiversity Research Centre, University of British Columbia, Vancouver, Canada.

## Abstract

Invasive species are a key driver of the global biodiversity crisis, but the drivers of invasiveness, including the role of pathogens, remain debated. We investigated the genomic basis of invasiveness in *Ambrosia artemisiifolia* (common ragweed), introduced to Europe in the late 19th century, by resequencing 655 ragweed genomes, including 308 herbarium specimens collected up to 190 years ago. In invasive European populations, we found selection signatures in defense genes and lower prevalence of disease-inducing plant pathogens. Together with temporal changes in population structure associated with introgression from closely related *Ambrosia* species, escape from specific microbial enemies likely favored the plant’s remarkable success as an invasive species.

## Teaser

The invasive success of ragweed in Europe was facilitated by release from enemy microbes and interspecies hybridization.

## INTRODUCTION

The wide-scale introduction of exotic species to novel ranges around the world can be largely attributed to the 19th century colonial activities of Europeans and to escalating global trade activities since the 20th century (*1*). Invasive species are now one of the major drivers of ecological change (*2*). They threaten global biodiversity and ecosystems by outcompeting native species (*3*, *4*). They also have a large economic impact due to damage (e.g., yield loss) and control measurements, with an estimated global cost of at least 1.288 trillion U.S. dollars between 1970 and 2017 (*5*). Attempts to stymie the rate of new introductions have failed on a global scale, as the rate of new introductions worldwide has not slowed down and may even be accelerating (*1*).

Many more species are introduced to novel ranges than become invasive. One of the fundamental questions in invasion biology is why some aliens become invasive, while others fail even to establish a permanent population (*6*). Some hypotheses attempt to explain the differential success of invasive species in relation to their traits. For example, certain characteristics of some plants (“ideal weeds”) make them more prone to become invasive, including prolific production of long-lived seeds, the rapid growth of seedlings, no biological necessity for specialized pollinators, self-compatibility, and adaptations for long-distance dispersal (*7*, *8*). Phenotypic plasticity may also be beneficial as fitness may be maintained under unfavorable environmental conditions or even increased under favorable conditions (*9*). In addition, there is a growing number of examples of rapid adaptation to the introduced environment that may facilitate invasion success (*9*–*13*). Selection in the introduced range is more likely to happen from standing genetic variation as new mutations are slow to arise. High genetic diversity in the introduced range is therefore beneficial. This can be facilitated through multiple introductions from diverse source populations and subsequent mixing in the introduced range (*9*). The introduction of species into new environments can also bring previously allopatric species into contact where they may hybridize. Hybridization may be a threat to species because of demographic and genetic swamping, but it can also be beneficial through adaptive introgression (*9*, *14*) and may help overcome the Allee effect in the early stages of invasion (*15*). Other hypotheses explaining species’ differential success as invaders invoke changes in ecological interactions in the introduced range, including escape from herbivores and pathogens [enemy-release hypothesis (ERH) (*16*, *17*)]. In plants, it has been proposed that the escape from specialized enemies in the native range allows an exotic species to allocate resources from defense mechanisms toward growth and reproduction, increasing its competitiveness in the introduced range [evolution of increased competitive ability (EICA) (*16*, *18*)].

Most studies so far focused on the release of herbivorous animals, but exotic introduction may also free plants from fungal (*19*), bacterial (*20*), oomycete (*21*), or viral (*22*) pathogens that live on their surface or within their tissues. Plants introduced to new geographic regions will also face new microbial interactions (*23*) that affect the plant’s fitness and, in some cases, could facilitate invasive success (*24*–*26*). So far, few studies have investigated the influence of microbial communities on the success of invasive plants and never at the genomic level. Considering the important role of plant-microbe interactions in evolutionary ecology (*27*), a characterization of the invasion process should also investigate the interplay of the host genome with its associated microbial metagenome (*28*–*31*).

To investigate the evolutionary genomic basis of plant invasion, we chose *Ambrosia artemisiifolia* (common ragweed), an extraordinarily successful noxious weed that is native to North America with a 200-year history of global introductions (*32*, *33*). It ranks as the 12th “worst” exotic plant in Europe, its impact assessed according to a panel of environmental and socioeconomic impact categories (*34*), and has established invasive populations in >30 European countries (*35*). *A. artemisiifolia* causes increasingly negative economic and public health impacts (*36*), mostly owing to its prolific production of highly allergenic, windborne pollen (*37*). Its future success is linked to climate change; thus, it is predicted to become a more serious problem in the coming decades (*38*, *39*). As shown by previous studies, *A. artemisiifolia* is able to rapidly adapt to its new environment (*40*–*43*) and thus has great potential to expand and become invasive in more regions. Recent work estimating the potential impact of biological control with the ragweed leaf beetle (*Ophraella communa*) offers some hope for reducing ragweed’s impact in Europe (*36*) and an indication that understanding ragweed’s ecological interactions may be one key to success in slowing the plant’s invasion.

*A. artemisiifolia* was introduced to Europe in the late 19th century and subsequently became invasive (*35*). It already occurred in botanical gardens in France as early as 1763, but it is believed that its spread was not caused by garden escapes (*32*). Two other species of the genus were also introduced to Europe, giant ragweed (*Ambrosia trifida*) and western ragweed (*Ambrosia psilostachya*) (*44*), but both are less abundant than *A. artemisiifolia* (*45*). Since its introduction to Europe, *A. artemisiifolia* has increased its abundance and range (*32*, *45*, *46*). Today, it is most widespread in France, Hungary, Serbia, and The Netherlands (*45*). We here use historic herbarium samples and contemporary samples from the native North American (NA) range and the introduced European range to (i) compare the two ranges over time and identify likely source populations for the European invasion, (ii) investigate whether there are fewer pathogens in the introduced range in accordance with ERH, and (iii) identify genomic regions involved in diverged selection in Europe over time and between Europe and North America.

## RESULTS

To uncover the process of invasion in this exceptionally successful invasive plant, we report a de novo assembly and annotation of the nuclear genome of common ragweed with a further analysis of 655 temporally sampled individual genomes and metagenomes from the native NA and the introduced European range (table S1 and fig. S1). Nearly 50% of these samples come from historical herbarium collections collected between 1830 and 1973. Historical herbarium samples show characteristics of ancient DNA damage: The C to T base misincorporation at the first base ranges from 1.1 to 9.6% (mean, 2.4%), and the endogenous content ranges from 55 to 96% (mean, 89%) (table S2). We grouped samples into five populations based on geography and genetic clustering. We found that the main source of the introduced European invasive population is the native-range Mideast population that was previously found to be of admixed origin (*47*) and likely arose because of the anthropogenic activities of early European colonists in North America.

We found large temporal changes in population structure in Europe, but not in NA, with several genetic clusters being exclusive to modern Europe. All spatial groups in Europe show signals of introgression from closely related *Ambrosia* spp. Metagenomic analysis revealed the presence of different plant pathogens, and differences between the native and introduced range were found. In Europe, we found evidence of recent selection on genes associated with defense, plant growth, and flowering time and differences in the presence and prevalence of plant pathogens between Europe and North America, consistent with changes in the composition of enemies as would be predicted by the ERH.

### De novo assembly of nuclear genome

Using the de novo assembly software Meraculous (*48*, *49*), an initial assembly of the short-read data from individual AA19_3_7 resulted in an assembly of length 1579.1 million base pairs (Mbp), composed of 93,647 scaffolds with an *N*_50_ of 89.7 kilo–base pairs (kbp). After filtering this initial assembly to remove haplotigs, the resulting filtered assembly consisted of 41,017 contigs with a total length of 1280.34 Mbp and an *N*_50_ of 101.591 kbp. After HiRise scaffolding with the Chicago sequencing data, the final genome assembly’s length was 1258.37 Mbp, and it was composed of 12,228 scaffolds with a scaffold *N*_50_ of 270.6 kbp. The repeat analysis resulted in an annotation of 30.76% of the genome sequence in interspersed repeats. Of the whole genome, 8.01% are long terminal repeat elements, 2.01% are long interspersed nuclear elements, 3.75% are DNA transposable elements, 0.20% are short interspersed nuclear elements, and 16.79% are unclassified repeats. The gene annotation of the repeat-masked genome resulted in 34,066 predicted proteins. The benchmarking universal single-copy orthologs (BUSCO) (*50*) analysis of assembly completeness determined the state of 255 single-copy ortholog genes and found that 136 (53.3%) were complete and single-copy, 101 (39.6%) were complete and duplicated, 15 (5.9%) were fragmented, and 3 (1.2%) were missing.

### Spatiotemporal population structure

**Fig. 1.**
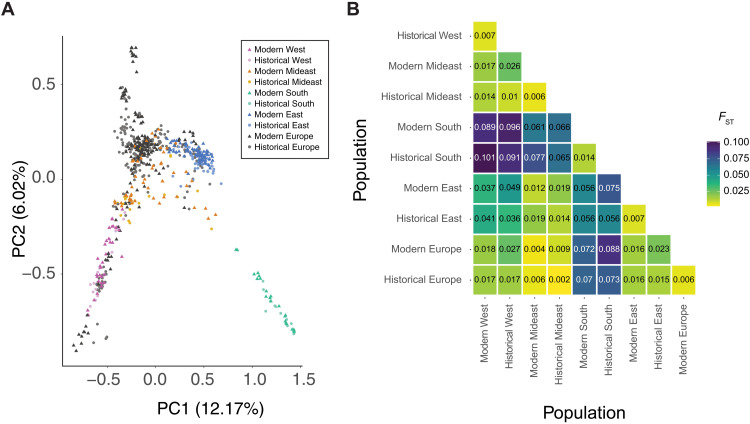
Population structure in *A. artemisiifolia*. (**A**) PCA of *A. artemisiifolia* samples. NA populations are defined on the basis of genetic clustering and geography. Dark pink, modern West NA; light pink, historical West NA; dark orange, modern Mideast NA; light orange, historical Mideast NA; dark turquoise, modern South NA; light turquoise, historical South NA; dark blue, modern East NA; light blue, historical East NA; dark gray, modern Europe; light gray, historical Europe. Circles, historical herbarium samples; triangles, contemporary samples. (**B**) Genetic structure estimated by pairwise *F*_ST_ (weighted) between native-range populations and Europe. Populations are split by time period (historical and modern). Shading of the boxes corresponds to the *F*_ST_ value, with yellow boxes indicating low *F*_ST_ and purple boxes indicating high *F*_ST_.

**Fig. 2.**
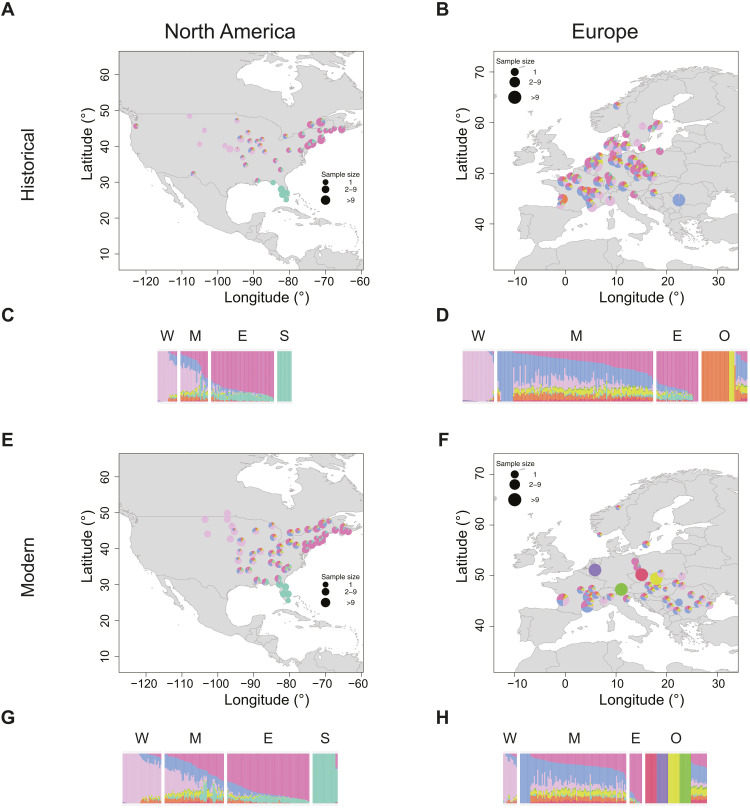
Admixture proportions for *A. artemisiifolia* populations. The NGSadmix run of a joint analysis including all samples with the highest likelihood for *K* = 9 was used for plotting. The same color scheme was used across all panels. (**A**, **B**, **E**, and **F**) Admixture maps. Samples within 100 km were grouped together, and the average ancestry across those groups was plotted. If samples were grouped together, then ancestry values were plotted at the centroid of the group. (**C**, **D**, **G**, and **H**) Admixture barplots. Each bar represents one individual. Samples are grouped on the basis of their geography: W, West; M, Mideast; E, East; S, South; O, other. (A) Historical North America. (B) Historical Europe. (C) Historical North America. (D) Historical Europe. (E) Modern North America. (F) Modern Europe. (G) Modern North America. (H) Modern Europe.

In the native NA range, samples cluster based on geography in both the principal components analysis (PCA) (Fig. 1) and admixture (Fig. 2) analysis, although genetic differentiation as measured by *F*_ST_ is low between these populations (Fig. 1). There are four main genetic clusters observed for *K* = 9 in the native range: the light pink cluster that is the main component of NA West samples, the dark pink cluster that is the main component of samples from NA East, the light turquoise cluster that is the main component of samples from NA South, and a blue cluster that is found in the Mideast samples that is located between the three extremes of the species range in NA (Fig. 2). The Mideast population has previously been shown to be of admixed origin and formed approximately 220 years ago (*47*). The geographic clustering in the native range did not change substantially between historical (collected between 1844 and 1939) and modern (collected between 2009 and 2019) times, and *F*_ST_ values are low when comparing the same populations through time (mean *F*_ST_ = 0.009; Fig. 1). The highest *F*_ST_ values can be observed in comparisons with the NA South population; although it is overall the most divergent, it is more similar to the NA East than to the NA West population. The South population contributed little to the Mideast population as evidenced by a clear separation of the South population from the other populations in the PCA, admixture component proportions, and high *F*_ST_ values. The Mideast population is located between the East and West population on the two first components of the PCA, connecting the two clusters and leading to a continuous distribution rather than a clear separation of the clusters. In the historical time period, it is closer to the West population, with the *F*_ST_ value being about twice as high between Mideast and East than between Mideast and West. In the modern time period, this difference disappears, with *F*_ST_ between Mideast and East being nearly identical to *F*_ST_ between Mideast and West. On the basis of *D*-statistics, we found evidence of introgression from the related species *A. trifida* in spatial groups from the West population. We also found evidence of introgression from *A. psilostachya* in spatial groups from the South and West populations based on *D*-statistics (figs. S2 and S3). Pairwise sequentially Markovian coalescent (PSMC)–based demographic reconstruction of a high-depth NA sample from the East population shows a population decline between 10^5^ and 10^4^ years ago (fig. S4).

**Fig. 3.**
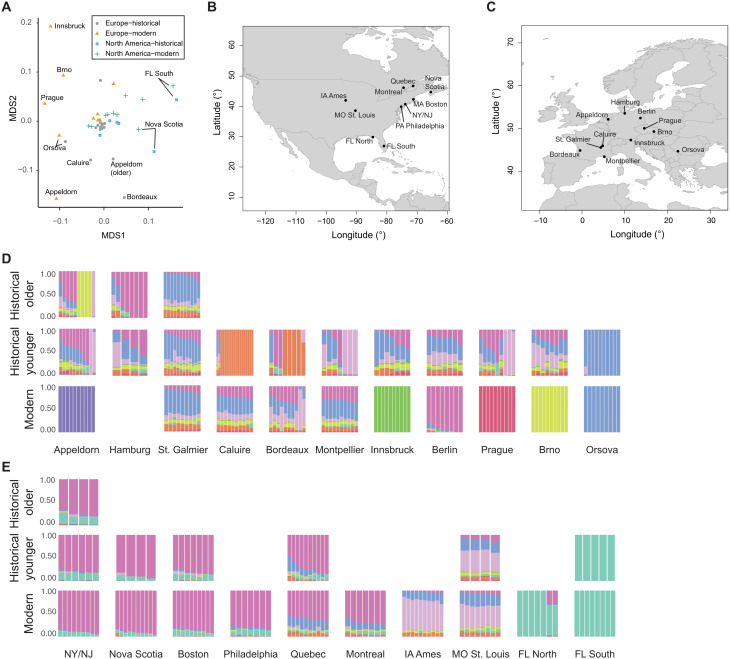
Genetic structure of spatial groups. (**A**) MDS plot of pairwise *F*_ST_ between spatial groups (stress = 0.17). Gray circles, historical Europe; orange triangles, modern Europe; blue squares, historical North America; green crosses, modern North America. (**B**) Location of spatial groups in North America. (**C**) Location of spatial groups in Europe. (**D**) Admixture barplots for *K* = 9 of European spatial groups. (**E**) Admixture barplots for *K* = 9 of NA spatial groups. If the spatial group was split into an older (collected before 1900) and younger (collected after 1900), then the top row shows the older historical time period, and the middle row shows the younger historical time period. Otherwise, the middle row shows the historical time period. Bottom row: Modern time period. For the admixture barplots, the same color scheme as in Fig. 2 was used. Admixture barplots are from a joint analysis run including all samples and were split into different panels.

In the introduced European range, no clear relationships with a single native-range population are observed in both historic and modern times. European samples do not cluster with the main NA South cluster on the PCA (Fig. 1) and show, overall, little of the main NA South cluster in the admixture analysis. None of the European samples were assigned to the South population based on the admixture analysis for *K* = 9. Furthermore, *F*_ST_ values are higher between Europe and NA South than between Europe and the other three NA populations. On the basis of *F*_ST_, Europe is almost equally close to the West and East population in both the historical and modern time periods. Europe shows the lowest genetic distance to the Mideast population, which is lower than values between the two time periods within the same populations. In the admixture analysis, more than half of the European samples for both the historical and the modern time periods are assigned to the Mideast population. The fraction of samples stays almost constant over time, with 56.7% in the historical time period and 54.7% in the modern time period. For both the East and the West population, the fraction of European samples assigned to them decreases over time. In historical times, 15.2% of samples were assigned to the East population and 11.4% to the West population. Among modern samples, only 6.5% were assigned to the East population and 7.1% to the West population. Over time, more samples within Europe could not be assigned to any NA population (historical time period: 35 samples, 17%; contemporary time period: 54 samples, 32%), and several unique genetic clusters are found in modern Europe (Fig. 2). These changes are evident in the analysis of spatial groups that show drastic changes over time in the admixture analysis (Fig. 3), with admixture diversity scores (table S3) decreasing in 6 of 10 European spatial groups, and 4 become unique genetic clusters not found in the native range. In the analysis of pairwise *F*_ST_, several modern European spatial groups cluster outside the NA range in the multidimensional scaling (MDS) analysis (Fig. 3B). Moreover, these spatial groups also cluster outside the NA range on the PCA (Fig. 1) and show low nucleotide diversity, low heterozygosity, and low effective population size (*N*_e_) (table S3). All spatial groups in Europe show signals of introgression from *A. trifida*, with the highest values found in those that form unique genetic clusters (Appeldorn, Innsbruck, Prague, and Brno) (fig. S2). In addition, two modern populations (Appeldorn and Bordeaux) show signals of introgression from *A. psilostachya* (fig. S3). Because of the relatively high genetic differences of geographically close spatial groups in Europe, no isolation-by-distance (IBD) pattern could be found in modern Europe, unlike in the native NA range (fig. S5). In historical Europe, a low *P* value slightly above the significance threshold (0.051) indicates weak IBD (*R*^2^ = 0.3). These changes in the IBD pattern in Europe support our findings of strong genetic changes in Europe over time. For those spatial groups that form unique genetic clusters in Europe, nucleotide diversity decreased over time, and the lowest value (0.0199) is found in modern Innsbruck (table S3). In addition, Tajima’s *D* substantially increases in these spatial groups over time, and the value changes from negative to positive in two of these (Appeldorn and Innsbruck). In historical Caluire and Bordeaux, a unique genetic cluster is found that is not found in modern times. These spatial groups show lower genetic diversity and have a lower admixture diversity score in the historic time period compared to the contemporary time period. The admixture diversity score of Montpellier also decreases over time, and no samples are assigned to the West population anymore (Fig. 3). However, the heterozygosity remains unchanged, and the nucleotide diversity decreases over time in this spatial group (table S2).

**Fig. 4.**
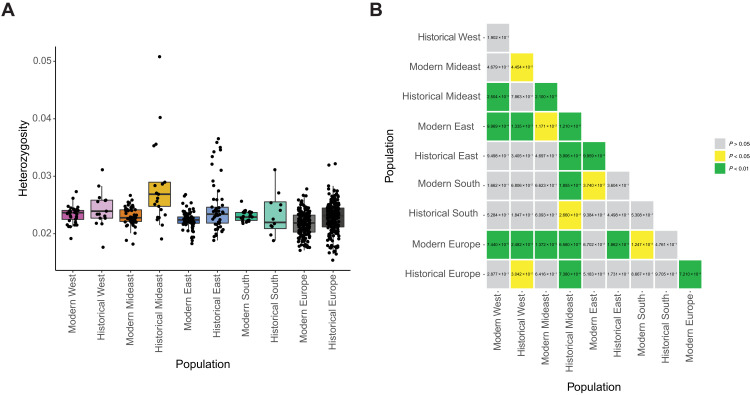
Heterozygosity. (**A**) Heterozygosity of individuals within NA and European populations. NA populations are defined on the basis of genetic clustering and geography. Color scheme is the same as in the PCA (Fig. 1). Samples with nuclear genome coverage below 0.5× after MAPQ 25 filtering were removed as downsampling experiments showed that the heterozygosity estimate at such low coverage diverges from the true heterozygosity (see the Supplementary Materials). (**B**) *P* values of Mann-Whitney *U* test between heterozygosity levels of different populations. Samples with coverage below 0.5× are removed. *P* values below 0.05 are highlighted in yellow, and *P* values below 0.01 are highlighted in green.

The highest mean heterozygosity is found in the historical Mideast population and is significantly higher (Mann-Whitney *U* test, *P* < 0.05) than in all other populations except historical West (Fig. 4). The lowest mean heterozygosity is found in the modern Europe population and is significantly lower (*P* < 0.05) than in all other populations except historical South and modern East. The lowest heterozygosity in the native range is found in modern East, which significantly differs (*P* < 0.05) from all other native-range populations except historical South. The effective population size (*N*_e_) is higher in Europe than in any of the NA populations (table S4) and decreases over time. In the native range, the South population has the lowest and the Mideast population the highest *N*_e_. In the native range, *N*_e_ increases over time for all but the East population. Tajima’s *D* is negative in all populations, with the lowest value found in historical Europe, followed by historical East and historical Mideast (table S4), and is generally lower in historical populations than in modern ones. All spatial groups except modern Appeldorn and modern Innsbruck have a negative Tajima’s *D* (table S3).

### Selection scanning

To determine the genomic regions where the European population experienced divergent selection from the NA population, we performed an analysis of *F*_ST_ outliers. To investigate whether these differences are due to changes over time or, rather, whether the initially introduced population already contained the differentiated genotypes and to exclude cases for which only the native-range population diverged over time, we additionally identified *F*_ST_ outliers in a comparison of the historical and modern European populations.

**Fig. 5.**
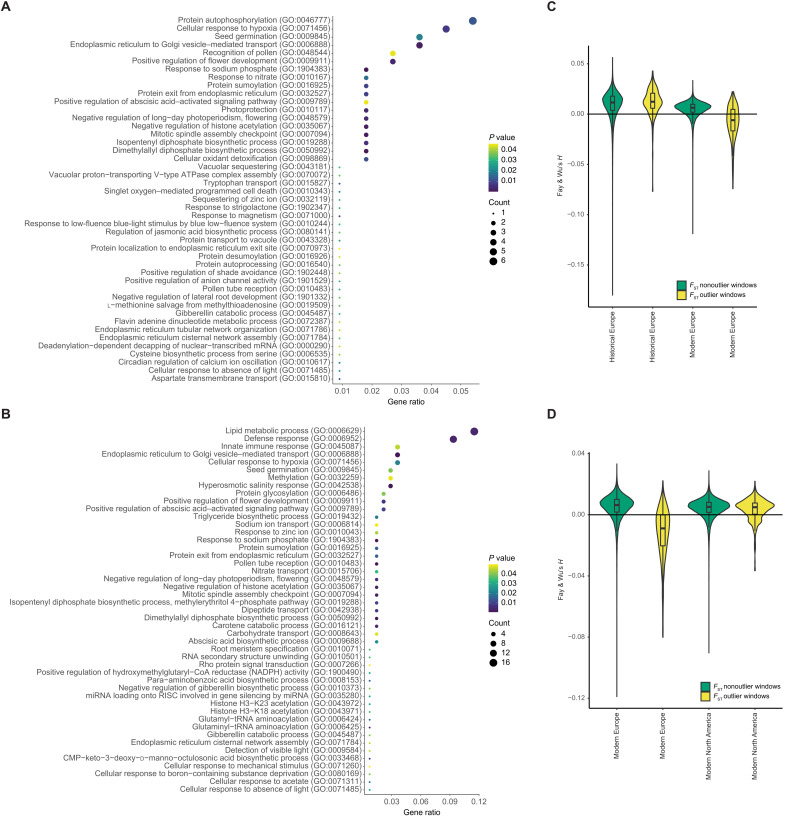
Selection scan. (**A** and **B**) Significantly enriched GO terms in the *F*_ST_ outlier windows. The size of the circles represents the number of significant genes annotated for the respective GO term. The color represents the *P* value for the enrichment, with yellow representing high and purple low *P* values. The gene ratio is the number of genes with significantly enriched GO terms in *F*_ST_ outlier windows divided by the total number of genes in *F*_ST_ outlier windows. (A) Enriched GO terms for historical versus modern Europe. (B) Enriched GO terms for modern Europe versus modern North America. (**C** and **D**) Fay and Wu’s *H* in *F*_ST_ outlier (yellow) and nonoutlier (green) windows. The boxplots show the median and first and third quantiles. (C) Outlier windows for *F*_ST_ between historical and modern Europe. (D) Outlier windows for *F*_ST_ between modern Europe and modern North America.

Between historical Europe and modern Europe, a total of 353 (0.38%) *F*_ST_ outlier windows were identified. These contained 111 *A. artemisiifolia* genes, of which 6 are orthologs of flowering time genes of *Arabidopsis thaliana* (*51*). Comparing the modern NA and European populations, a total of 442 (0.43%) *F*_ST_ outlier windows were found. These contained 139 unique genes, of which 7 were homologs of *A. thaliana* flowering time genes (*51*). Of the outlier windows, 159 are shared between the comparisons of historical/modern Europe and modern Europe/modern NA (fig. S6). To infer whether the *F*_ST_ outlier windows were under positive selection in Europe, we estimated Fay and Wu’s *H* (*52*) for each window at each time point. We did this with the expectation that *F*_ST_ outlier windows would show lower estimates of *H* in modern Europe than nonoutlier windows if sweeps had occurred following introduction in these genomic regions. This neutrality test uses the ancestral state to infer the derived allele and is therefore less influenced by demography than Tajima’s *D*, and a negative *H* indicates positive selection. For both comparisons (modern Europe versus North America and modern Europe versus historic Europe), outlier windows have, on average, a negative *H* in modern Europe, which is significantly lower than in nonoutlier windows (*P* < 2.2 × 10^−16^), while outlier windows in historical Europe and modern NA show a positive Fay and Wu’s *H* on average (Fig. 5).

The gene ontology (GO) enrichment analysis shows 45 enriched GO terms in the comparison of historical and modern Europe (Fig. 5 and table S5). Among other functions, these GO terms are associated with growth, stress response, light response, circadian regulation, response to phosphate and nitrate, flowering, and pollen recognition. Of these, 17 are also shared in the comparison of modern Europe and North America. Between modern Europe and modern North America, 47 GO terms are enriched in *F*_ST_ outlier windows (Fig. 5 and table S5). These include functions associated with growth, defense, response to salinity, flowering, and response to phosphate. Of the top outlier single-nucleotide polymorphisms (SNPs; *Z* > 50) between historical and modern Europe, 43 of 414 are found within 23 gene regions (table S6). Several of these genes are orthologs of well-characterized genes in *A. thaliana*. For example, functional analysis of AT1G47740 (PPPDE putative thiol peptidase family protein) in *A. thaliana* showed that mutants are involved in abiotic stress response evidenced by expression changes in response to cold (down-regulation), oxidation (down-regulation), and osmotic stress (up-regulation) (*53*). Mutants of AT2G13540 [*ABA HYPERSENSITIVE 1* (*ABH1*)] are early flowering (*54*), drought resistant (*55*, *56*), and hypersensitive to the plant hormone abscisic acid, which regulates development and stress response (*55*). AT5G47910 [*RESPIRATORY BURST OXIDASE HOMOLOGUE D* (*RBOHD*)] is involved in defense response to abiotic stress and pathogens (*57*, *58*), specifically via its interaction with the *AtrbohF* gene, which allows tuning the spatial control of the production of reactive oxygen intermediates and hypersensitive response around sites of infection (*58*).

### Pathogen identification

**Fig. 6.**
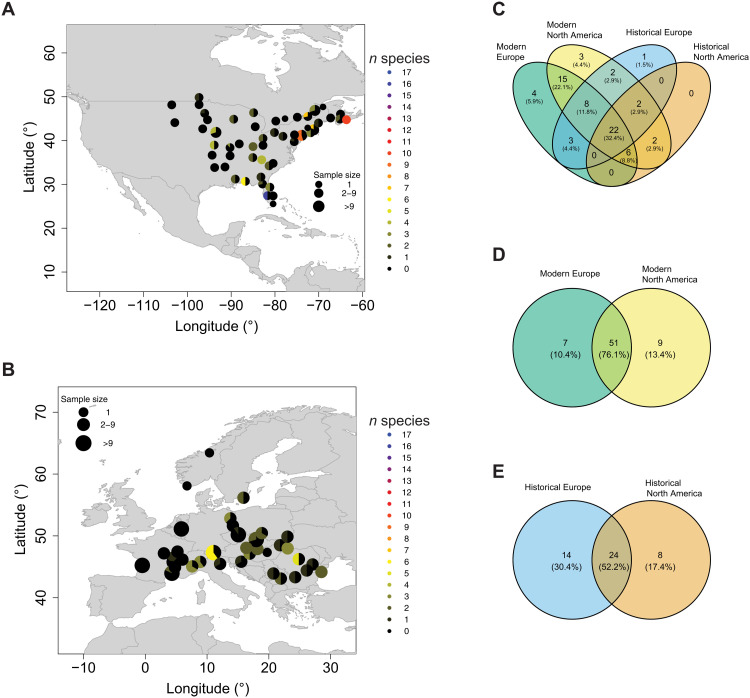
Pathogen presence. (**A** and **B**) Prevalence of *Xanthomonas* spp. in contemporary samples. Samples within 100 km were grouped together. The pie chart indicates the fraction of samples in which *Xanthomonas* spp. are present, with black indicating no *Xanthomona*s species identified. The color indicates how many different *Xanthomonas* species were identified at a location. (A) Modern North America. (B) Modern Europe. (**C**) Venn diagram of pathogens identified in modern European (green), modern NA (yellow), historical European (blue), and historical NA (orange) samples. (**D**) Venn diagram of pathogens identified in modern European (green) and modern NA (yellow) samples. (**E**) Venn diagram of pathogens identified in historical European (blue) and historical NA (orange) samples.

We classified the metagenomes of 305 historical herbarium and 350 contemporary leaf samples. Only species that were previously described as plant pathogens in the FAPROTAX database (*59*) were considered. We identified a total of 68 different pathogens in the entire dataset (Fig. 6 and table S7). Fewer pathogens were identified in the historical herbarium samples (38 in Europe and 32 in North America) compared to the contemporary samples (58 in Europe and 60 in North America). This difference could result from the shorter fragment length and ancient DNA damage in the historical samples, which could reduce the detectability of historic pathogens. Moreover, historical herbarium specimens can be colonized by microbial taxa during preservation or storage in the herbarium (“herbarium contamination”) (*60*). As this makes it challenging to distinguish the natural microbial community from herbarium contamination, comparisons were only done within time periods. In the historical time period, 8 pathogen species detected in NA are absent in Europe, while 14 pathogens detected in Europe are absent in NA. Five species have a significantly higher (*P* < 0.05) prevalence in historic Europe, while one is significantly higher (*P* < 0.05) in historical NA. In the contemporary samples, nine pathogens are absent from Europe and present in NA. Of these, two show the same pattern in the historical time period. Seven pathogens present in modern Europe are absent in modern NA. Of these, three show the same pattern in the historical specimens. In the contemporary time period, 8 pathogens show a significantly higher (*P* < 0.05) prevalence in Europe and 12 in North America. In general, either more or a significantly higher prevalence (*P* < 0.05) of *Xanthomonas* (Fig. 6) and *Pseudomonas* taxa is found in North America, and either more or a significantly higher prevalence (*P* < 0.05) of *Dickeya* and *Brenneria* species is found in Europe. Of the 17 plant pathogen bacteria *Xanthomonas* species identified in our dataset, only 2 species were significantly more abundant (*P* < 0.05) in ragweed populations in modern Europe than in modern North America, and 2 more were completely absent from modern Europe (table S5). In the historical period, 8 of these *Xanthomonas* species were absent from North America, and 12 were absent from Europe. In modern Europe, *Dickeya* species are found throughout the whole range, while *Xanthomonas* spp. are more common and *Brenneria* spp. are restricted to south-eastern Europe (Fig. 5 and fig. S7).

## DISCUSSION

Using the largest collection of conspecific genomes derived from herbarium specimens of any species, we found a remarkable turnover in the genetic structure of introduced *A. artemisiifolia* populations over the brief window of time during which this plant has established itself in Europe. This finding likely reflects multiple introductions from diverse sources, drift during introduction bottlenecks, and even introgression from related *Ambrosia* species. Moreover, we found evidence of rapid adaptation and a pattern in the presence of microbial pathogens (*Xanthomonas* spp.), consistent with the enemy escape hypothesis, but here, more validation is needed. This indicates that multiple factors such as admixture of different source populations, hybridization with related species, and escape from pathogens facilitated the success of *A. artemisiifolia* in Europe.

Using high-resolution spatiotemporal sampling of *A. artemisiifolia* populations in both NA and Europe, we found genomic signals of putative divergent selection during range expansion, in combination with previous work (*41*, *61*), this points to rapid adaptation during the invasion. Many of these associated genes were involved in responses to stress and light, as well as flowering, defense, and growth. Our results provide a genomic foundation for understanding previous work describing major phenotypic differences between samples from Europe and the native ranges, with European populations characterized by reduced drought resistance and a higher allocation of resources toward growth and reproduction (*41*), well in line with earlier common garden experiments that showed strong evidence of adaptation in the European range in traits such as plant size, reproduction investment, sex allocation, phenology, dichogamy, specific leaf area, and plant growth (*61*). Adaptive processes may have also caused differences in early life cycle stages in European and NA populations. Germination rate, germination speed, and frost tolerance of seedlings have significantly diverged between the ranges, and temperature niche width for germination is significantly broader for European populations (*62*). Consistent with our finding of rapid adaptation of flowering genes, European populations already show clines in flowering time, similar to those found in the native range, which likely reflect local adaptation (*43*).

It has long been suggested that escape from natural enemies in the native range can facilitate the invasion success of introduced species (*16*–*18*). These enemies can not only be animals like the ragweed leaf beetle (*O. communa*) but also any of the multitudes of microbial pathogens known to affect *A. artemisiifolia* (*63*). In addition to some plant pathogen bacteria being absent or of lower prevalence in Europe (e.g., *Xanthomonas*), we also detected some taxa only in Europe or at higher prevalence than in North America (e.g., *Brenneria* and *Dickeya*). Bacterial plant pathogens often have high host specificity but can sometimes live on nonhost plants without causing disease (*64*). It is thus important to ascertain whether the particular non-*Xanthomonas* pathogens actually cause disease in *A. artemisiifolia* before drawing conclusions about enemy escape. *Brenneria*, *Dickeya*, and *Xanthomonas* are the three genera that show differences in prevalence or presence between Europe and North America. Of these, only *Xanthomonas* spp. are known to cause disease in *A. artemisiifolia*, inducing 60% mortality in infected plants (*64*), and show reduced prevalence in Europe. Both *Brenneria* and *Xanthomonas* are absent or at very low prevalence in France where *A. artemisiifolia* was first introduced to Europe. This could have helped *A. artemisiifolia* to establish a stable population in France and its subsequent spread. These findings are in favor of the ERH and suggest that, together with the release from specialist herbivores such as the ragweed leaf beetle, escape from some native-range microbial plant pathogens may have facilitated the success of *A. artemisiifolia* in Europe. The change in pathogen composition, including the addition of novel species, in Europe compared to NA likely induced selection on defense genes in Europe. We find genes associated with defense response enriched in *F*_ST_ outlier windows when comparing the modern native and introduced range. These windows also show signatures of recent positive selection in modern Europe (Fig. 5). We find an *F*_ST_ outlier SNP in the gene *RBOHD*, which is associated with defense response and response to wounding, when comparing historical and modern Europe. The window containing this gene has a sweep signature in modern Europe indicated by a negative Fay and Wu’s *H*. Further work should be done to characterize the role of the *RBOHD* gene and other defense genes putatively under selection in Europe in the immune response of *A. artemisiifolia*.

The population structure we found in the native range reinforced previous results based on reduced-representation genomic data from contemporary samples (*47*, *65*). The most differentiated population is the South population in North America, which is restricted to the southeastern United States (Florida, coastal Mississippi, and Georgia). On the basis of the palynological records from sediment cores (*66*, *67*), this region was a refugium during the Last Glacial Maximum. If the East and West populations originate from different glacial refugia, then this could explain the higher divergence of the South population from other NA populations. The South population has the lowest *N*_e_ in both historical and modern times. Together with the negative Tajima’s *D* and admixture results, this indicates that the South population experienced a population expansion but stayed relatively isolated from the other NA populations. On the basis of the global *F*_ST_, PCA, and admixture results, this population did not contribute substantially to the Mideast population in the native range or to the introduced European range.

For NA, in contrast to Europe, we found clear stasis in the population genetic structure. The only exception to this was a shift in the extent of the Mideast population. On the basis of simulations using approximate Bayesian computation based on random forest algorithms (ABC-RF) and reduced-representation genomic data from present-day populations, van Boheemen *et al.* (*47*) estimated that the Mideast population formed through admixture of the East and West populations more than 200 years ago, thus suggesting that it predated the introduction of the species to Europe. Anthropogenic disturbances such as forest clearance and the expansion of agriculture are the most likely cause for the formation of this admixed population (*33*). By directly sampling historical herbarium samples, we confirm that this cluster already existed in the late 19th century, with the oldest sample in our study from this population dated to 1875. High heterozygosity values and a relatively high *N*_e_ for the historical Mideast population, especially compared to the modern Mideast population, indicate that the admixture event happened shortly before the period in which most of our historical samples were collected.

Despite being present in French botanical gardens as early as 1763, wild populations of common ragweed were not reported before the late 19th century in France (*32*) and even later in other parts of Europe (*68*). Historical records and herbarium data suggest that there were several independent introductions of *A. artemisiifolia* into Europe, rather than a single introduction and a subsequent spread (*32*). These introductions likely arose from different source populations, as we find that many historical samples from Europe are fully assigned to a native-range population (11% West, 15% East, and 57% Mideast). Some samples (17%) could not be assigned to any of the native populations, and the fraction of these samples increases over time to 32%. This suggests that *A. artemisiifolia* was already present in Europe well before most of our historical samples were collected and that genetic drift associated with initially small population sizes indicated by a decrease in nucleotide diversity and *N*_e_, combined with strong selection pressure, may have led to the rapid formation of unique genetic clusters early in Europe.

It is also clear that introgression from related plant taxa contributed to the formation of these unique genetic clusters in Europe. *A. artemisiifolia* can produce hybrids with *A. trifida* (*69*) and *A. psilostachya* (*70*), both of which are native to North America and were introduced to Europe around the same time as *A. artemisiifolia* (*71*). They thus had opportunities to hybridize with *A. artemisiifolia* in the introduced range, although hybrids have not yet been reported to produce viable seeds (*35*). We find signals of introgression from both *A. trifida* and *A. psilostachya* in Europe with high levels in those populations that form unique clusters in Europe. Because of the lack of compatible mates during the early stages of invasion when population sizes are low, species may not be able to establish a population (Allee effect). Hybridization in the introduced range may mitigate Allee effects, which tend to be particularly strong in self-incompatible species such as *A. artemisiifolia* (*72*) and can thus facilitate invasion success even if the introgressed regions are not adaptive (*15*). Previous studies found that introgression is expected from a native or previously established species toward the introduced species (*73*). We here, however, found evidence for introgression from two other introduced species that arrived in Europe around the same time as *A. artemisiifolia* but are less successful. It is possible that introgression was unidirectional toward *A. artemisiifolia* as hybrids may not be able to backcross with *A. trifida* and *A. psilostachya*, and this may be a reason for the higher invasive success of *A. artemisiifolia* compared to the two other *Ambrosia* species. In addition to “demographic rescue,” interspecific hybridization early in the invasion may have offered *A. artemisiifolia* populations other benefits, including heterosis and the adaptive introgression of beneficial alleles. As we also find introgression in some native-range populations, it is possible that the observed pattern in Europe is due to older introgression in the source population. Future studies should investigate the genome-wide pattern of introgression to identify introgressed regions and explore whether these are likely to be adaptive. Moreover, the temporal pattern of introgression should be further investigated to infer whether hybridization was more common in the early stages of the *A. artemisiifolia* invasion of Europe, when the species was rare.

The origin of the European *A. artemisiifolia* invasion has been debated in the literature. All studies have found evidence for multiple introductions (*32*, *40*, *74*). However, some studies have suggested that the admixture was largely sourced from the native-range admixed region (*33*), while others suggested that the admixture occurred after invasion (*74*, *75*). Our temporally stratified snapshots of population structure provide clearer insights into this debate.

As a whole, the European population is most closely related to the NA Mideast population, which is of admixed origin (*47*). Moreover, most of the European samples cluster with the Mideast population in the PCA, and more than half of the samples are assigned to this population in the admixture analysis. None of the samples collected before 1892 in Europe (*n* = 78) were assigned to the West cluster. According to herbarium data and bibliography, the first introductions to Europe occurred over a short time period of about 10 years from Eastern North America (*76*). We thus find it unlikely that substantial admixture between individuals that originated from the native-range East and West genetic cluster in the introduced range led to the observed pattern and conclude instead that the native-range Mideast cluster was the main source of introduction in Europe. The East and West populations also seem to have contributed to Europe, as several European samples not only show nearly complete assignment to the main West or main East genetic cluster in the admixture analysis but also group with the West or East population on the PCA. The prevalence of West and East cluster ancestry decreases over time in Europe, while the fraction of samples being assigned to the Mideast cluster does not. Previously, it has been suggested that the population genetic differentiation between eastern and western European *A. artemisiifolia* reflects historical introduction and trade routes (*77*). In contrast, our results indicate that the main source of introduction in both western and eastern Europe is likely the Mideast population from the native range. The oldest samples in both eastern and western Europe are frequently (57%) placed in the Mideast cluster, supporting the hypothesis that substantial admixture occurred either before introduction or very early in the invasion process. Because of the admixture of several native populations, much of the native-range genetic variance was introduced to historical Europe, as evidenced by no significant decrease (*P* > 0.05) in heterozygosity levels in Europe compared to the native range and higher *N*_e_ than in the native range. Over time, the European population has diverged from the NA population, as fewer samples are assigned to the native genetic clusters, and more genetic clusters unique to Europe have emerged.

The increasing pace of biological introduction to novel ranges via global trade and climate change severely threatens global biodiversity. Using the weed *A. artemisiifolia* as a model for plant invasion, we demonstrate that combining population genomic analysis with a metagenomic approach can identify factors that may facilitate the success of plant invaders both before and following the introduction event. These factors include preintroduction admixture of different source populations in the native range, rapid adaptation, introgression from other species, and the escape from some plant pathogens. We show that microbial pathogens both old and new play a role in the adaptive landscape of highly successful invasive plants such as *A. artemisiifolia*. The identification of *A. artemisiifolia*’s lost native-range pathogens informs future efforts to devise effective biological control measures.

Our study illustrates the potential of global herbarium collections as a rich source of historical material for high-resolution population genomic and metagenomic investigations over continental and even global spatial scales. These meticulously curated and often well-preserved plant specimens contain not only the host plant genome but also a complex community of associated microbes that, when considered together in a hologenomic framework, can reveal a rich history of coevolution and networks of synergy and antagonism during the Anthropocene. Our study highlights that multiple factors facilitated the invasion success of *A. artemisiifolia*. This is likely also the case for other invasive species. Future studies should thus use an integrated approach combining population genetics, metagenomics, and introgression analysis. Moreover, integration of other genomics such as epigenomes and transcriptomes can further elucidate the basis of successful invasions.

## MATERIALS AND METHODS

### De novo assembly of reference genome

Seeds were collected from a natural *A. artemisiifolia* population in North Dakota, USA (46.298° latitude, −103.918° longitude) in 2008. From these seeds, the individual plant AA19_3_7 was grown to maturity in a greenhouse at the Department of Botany, University of British Columbia. Fresh leaf tissue was sampled, flash-frozen in liquid nitrogen, and then stored at −80°C. High–molecular weight (HMW) genomic DNA was extracted using a Cetyltrimethylammoniumbromid (CTAB) protocol (*78*, *79*) and prepared for short-insert sequencing and mate-pair sequencing. Sequencing was performed at Genome Quebec, generating four lanes of short-insert data from two TruSeq libraries and two lanes of Nextera mate-pair data from two libraries (5- and 10-kbp inserts) on an Illumina platform. After adapter trimming with Trimmomatic v0.38 (*80*), the data were assembled using Meraculous-2D v2.2.6 (*48*) with the command-line options diploid_mode 2, no_strict_haplotypes 0, genome_size 2, gap_close_aggressive 1, mer_size 55, and min_depth_cutoff 11.

*A. artemisiifolia* is self-incompatible, so it was not possible to obtain a homozygous plant for sequencing and assembly of a reference genome. Because of the heterozygous nature of the genome, our initial assembly contained many near-duplicate haploid contigs (haplotigs) from individual AA19_3_7’s homologous chromosomes. We attempted to remove these haplotigs from the reference assembly to ensure that the length of the reference genome is not falsely inflated using the software PurgeHaplotigs (*81*). After producing a histogram of contig read depth, low, mid-point, and high cutoffs were designated as read depth values of 20, 110, and 195, respectively. Alongside a junk threshold of 80% (-j 80) and a suspected haplotig threshold of 60% (-s 60), these values were used to create a pool of suspect haplotigs. A final step was performed to split duplicate contigs into a haplotig pool using a threshold of 70% (-a 70) identity to confirm a contig as a haplotig and then to create a new, filtered assembly with the haplotigs removed. Comparison of basic single-copy orthologous (BUSCO) genes revealed a large increase in single-copy genes after purging, using BUSCO v5.1 (*50*). Initial BUSCO scores were 239 complete buscos (62 single copy, 177 duplicated, 12 fragmented, and 4 missing), while final BUSCO scores after purging were 237 complete buscos (136 single copy, 101 duplicated, 15 fragmented, and 3 missing) of 255 BUSCO groups searched. This was provided as the input assembly for the genome assembly scaffolding described below.

Additional HMW genomic DNA was provided to the commercial provider Dovetail Genomics, which prepared two Chicago libraries as described previously (*82*). Briefly, for each library, ~500 ng of HMW genomic DNA was reconstituted into chromatin in vitro and fixed with formaldehyde. Fixed chromatin was digested with Mbo I, the 5′ overhangs filled in with biotinylated nucleotides, and then free blunt ends were ligated. After ligation, cross-links were reversed, and the DNA was purified from protein. Purified DNA was treated to remove biotin that was not internal to ligated fragments. The DNA was then sheared to a ~350-bp mean fragment size, and sequencing libraries were generated using NEBNext Ultra enzymes and Illumina-compatible adapters. Biotin-containing fragments were isolated using streptavidin beads before polymerase chain reaction (PCR) enrichment of each library. The Chicago libraries were then sequenced on an Illumina HiSeq 2500 platform. The number and length of read pairs produced for each library were as follows: 256 million, 2 × 101 bp for library 1; 103 million, 2 × 101 bp for library 2. Together, these Chicago library reads provided 25.6× physical coverage of the genome (1- to 100-kbp pairs).

The input de novo assembly and Chicago library reads were provided to HiRise, a software pipeline designed specifically for using proximity ligation data to scaffold genome assemblies (*82*). Chicago library sequences were aligned to the draft input assembly using a modified SNAP read mapper (available from http://snap.cs.berkeley.edu). The separations of Chicago read pairs mapped within draft scaffolds were analyzed by HiRise to produce a likelihood model for genomic distance between read pairs, and the model was used to identify and break putative misjoins, to score prospective joins, and to make joins above a threshold. After scaffolding, shotgun sequences were used to close gaps between contigs.

### Annotation of reference genome

From sample AA19_3_7, we harvested each of four tissue types (leaf, stem, male flowers, and root) by placing the tissue into aluminum envelopes and then into liquid nitrogen. The frozen tissue samples were ground using a mortar and pestle, and total RNA was purified using commercially prepared TRIzol reagent. The total RNA extracts were visualized using a Bioanalyzer instrument using a total RNA 6000 chip. The Bioanalyzer generates an RNA integrity number (RIN) that assesses the integrity of the total RNA sample, and total RNA extracts passed quality control if the RIN was ≥7.5. The total RNA extracts from each tissue type were sent to the Genome Quebec Innovation Centre at McGill University where each one was prepared into a barcoded and stranded TruSeq mRNA library with an insert size of 165 bp (±10%). The four completed libraries were pooled in equal amounts and sequenced on one lane of an Illumina HiSeq 2000 instrument. The RNA sequencing data were first cleaned using Fastx (Hannon Lab), and then a de novo reference was assembled with all four libraries using Trinity (*83*).

Repeats and low-complexity DNA sequences were masked in the genome before gene annotation using RepeatMasker version 4.1.0 (*84*) using the species repeat database “Asteraceae” with Repbase database version 20170127. Remaining specific repetitive elements were predicted de novo using RepeatModeler version 2.0.1 (*85*) on the masked genome. Subsequently, a second round of RepeatMasker was run with the model generated from RepeatModeler as custom library input on the previously masked genome. Genome annotation was performed using the genome annotation pipeline MAKER2 version 2.31.9 (*86*) with ab initio and homology-based gene predictions. Unique protein sequences (3819) from asterids (a monophyletic group of flowering plants), Asteraceae (sunflower family), and *Ambrosia* (ragweeds) were used for homology-based gene prediction. As no training gene models were available for *A. artemisiifolia*, we used CEGMA (*87*) to train the ab initio gene predictor SNAP (*88*). MAKER2 was run with command-line arguments model_org=simple, softmask=1, and augustus_species=arabidopsis, and the snaphmm parameter was set to the hidden Markov model (HMM) generated in the manual training of SNAP. As expressed sequence tag evidence, we used the Trinity-assembled transcriptome. The 34,066 predicted proteins were then compared with *A. thaliana* annotations [TAIR 10 representative gene model proteins (*89*)] using the blastp command in BLAST+ (*90*). A total of 29,927 predicted *A. artemisiifolia* proteins matched *A. thaliana* genes with an *E* value of <1 × 10^–6^; these annotations were retained for downstream analysis. In addition, annotations were cross-referenced with 306 *A. thaliana* flowering time genes (*51*). A total of 566 predicted *A. artemisiifolia* genes were matched to this dataset, representing 191 unique *A. thaliana* flowering time genes.

### Acquisition of contemporary samples

For this study, we generated new shotgun sequencing data for 382 historical herbarium and 366 contemporary specimens of *A. artemisiifolia* and combined it with already published data (*60*, *91*). A total of 77 herbarium samples and 16 contemporary samples were later removed for various reasons (see sections below), leading to a final dataset of 655 samples. A full overview of sample sources, laboratory methods, and inclusion in the final dataset can be found in table S1. Sampling locations are displayed in fig. S1.

Silica-dried leaf tissue samples were obtained from wild populations in NA and Europe via the authors’ personal collections from previously published studies (*33*, *61*) and a network of collaborators. Some samples were provided as seeds, which were sown and raised in the greenhouse facilities at the Norwegian University of Science and Technology (NTNU) University Museum’s Ringve Botanical Garden (Trondheim, Norway) or at Monash University (Melbourne, Australia). From these plants, leaf tissue was harvested and silica-dried. See table S1 of sample provenance.

### Acquisition of historical herbarium samples

To summarize and select from available *A. artemisiifolia* herbarium specimens, we made a list of samples already available from previous studies (*33*, *41*, *47*), those we found in online databases of herbarium collections (e.g., the Global Biodiversity Information Facility; www.gbif.org), and by directly contacting herbaria. We preferred specimens that were collected before 1940 to represent the initially introduced populations into Europe. For some European regions (Romania and Czech Republic), no or very few herbarium samples from before 1940 were available. To include those regions, we used the oldest available herbarium samples instead (fig. S1). When the collection latitude and longitude coordinates were not available as metadata, this information was inferred from the centroid (as defined by the web tool Google Maps) of the most specific geographic sampling location (e.g., the city) described on the herbarium voucher sheet. A complete list of samples included in this study can be found in table S1, along with sample locations and assignment to broader populations (as described below). To evaluate whether specimens in our dataset were misidentified and to test for introgression of some species, we included shotgun sequencing data of 123 samples of other *Ambrosia* species (hereinafter referred to as outgroup samples), including samples of known hybrids of *A. artemisiifolia* with other *Ambrosia* species (table S8).

### DNA extraction, library preparation, and sequencing of herbarium samples

For the historical herbarium samples processed for this study, all pre-PCR steps were carried out in a dedicated, positively pressurized ancient DNA laboratory at the NTNU University Museum. DNA was extracted from leaf tissue using the QIAGEN DNeasy Plant Mini Kit according to the manufacturer’s instructions except for the addition of an overnight incubation step with proteinase K, as previously described (*33*). DNA concentration was quantified with a Qubit 2.0 fluorometer using the BR dsDNA kit. Extraction blanks were prepared alongside the samples to monitor possible contamination.

DNA extracts were converted into blunt-end double-stranded Illumina libraries using the BEST protocol (*92*), in which custom blunt-end adapters (*93*) were ligated to the DNA fragments or single-stranded Illumina libraries using the Santa Cruz Reaction protocol (*94*). During indexing PCR, custom index primers were used to generate dual-index libraries. Indexing PCR was carried out either in a 50-μl or a 100-μl reaction with 5 to 10 μl of library template, 0.2 mM each deoxynucleotide triphosphate (dNTP), 0.2 μM sample-specific forward index primer, 0.2 μM sample-specific reverse index primer, AmpliTaq Gold DNA polymerase (0.05 U/μl), 1× AmpliTaq Gold buffer, 2.5 mM MgCl_2_, bovine serum albumin (0.4 mg/ml), and the rest of the reaction volume filled up with molecular-grade water. The PCR was performed with an initial denaturation of 10 min at 95°C and then *X* cycles of a 30-s denature at 95°C, 1 min of annealing at 60°C, and 45 s of extension at 72°C, followed by a final extension of 5 min at 72°C. The optimal number of PCR cycles *X* was selected for each sample on the basis of quantitative PCR. Amplified libraries were purified with solid phase reversible immobilization (SPRI) beads (*95*) and eluted in 33 μl of QIAGEN EB buffer. For some samples, two indexing PCRs were performed to increase library complexity. Samples were pooled and sequenced on Illumina platforms (see table S1 for details about the sequencing platforms used).

### DNA extraction, library preparation, and sequencing of modern samples

For contemporary samples, leaf tissue was collected and stored in silica gel desiccants (*96*) at room temperature until required for DNA extraction. Approximately 20 to 30 mg of dried leaf tissue from each sample were placed inside a 2.0-ml tube with a 3-mm stainless steel bead and ground with a TissueLyser II (QIAGEN). The DNA was extracted using a modified CTAB protocol (*97*) adapted for a 96-well plate format (*98*) using EconoSpin filter plates, and the DNA was suspended in 60 μl of elution buffer. Extracted DNA was quantified using a Qubit 2.0 fluorometer (Invitrogen, Carlsbad, CA, USA) using the high-sensitivity dsDNA kit. Extraction blanks were prepared alongside the samples to monitor possible contamination.

Extracts were converted into blunt-end Illumina libraries as described above. Indexing PCR was carried out in a 100-μl reaction using 7.5 μl of DNA template, 0.2 mM each dNTP, 0.2 μM sample-specific forward index primer, 0.2 μM sample-specific reverse index primer, 1× Herculase Fusion II DNA polymerase, and 1× Herculase II reaction buffer and the remaining volume filled up with molecular-grade water or in a 50-μl reaction with 5 μl of library template, 0.2 μM sample-specific forward index primer, 0.2 μM sample-specific reverse index primer, and 1× Platinum SuperFi PCR master mix and the rest of the volume filled up with molecular-grade water. The PCR with Herculase was performed with an initial denaturation of 3 min at 95°C, followed by 12 cycles of a 20-s denaturation at 95°C, 20 s of annealing at 60°C, and 40 s of extension at 72°C, followed by a final extension for 5 min at 72°C. Amplified libraries were purified with SPRI beads (*95*) and eluted in 33 μl of EB buffer. The PCR with SuperFi was performed with an initial denaturation of 3 min at 98°C, followed by 12 cycles of a 20-s denature at 98°C, 1 min of annealing at 60°C, and 45 s of extension at 72°C, followed by a final extension of 5 min at 72°C. Amplified libraries were purified with SPRI beads and eluted in 33 μl of EBT buffer. Samples were pooled and sequenced on Illumina NovaSeq. In addition, 44 samples were sequenced on the DNBSEQ-G400 platform. See table S1 for details about the sequencing platform used.

### Sequence alignment to reference genome

Raw reads were processed with the paleomix v.1.2.13.8 BAM pipeline (*99*). AdapterRemoval v2.3.1 (*100*) was used to remove sequencing adapters, and reads with a minimum overlap of 11 bases were collapsed into one read and treated as single-end reads during the mapping. Sequences were aligned to the *A. artemisiifolia* reference genome assembly and against the *A. artemisiifolia* chloroplast reference genome (GenBank: MG019037.1) using bwa v0.7.17 mem (*101*) without filtering for quality. PCR duplicates were marked using either picardtools MarkDuplicates v2.21.2 (http://broadinstitute.github.io/picard) or bammarkduplicates from the biobambam2 v2.0.87 package. MapDamage2 (*102*) was used to calculate the frequencies of base misincorporation for historical samples. The paleomix summary files were used to obtain mapping statistics. The endogenous content was estimated as the fraction of raw reads mapping against the *A. artemisiifolia* reference genome. Statistical analysis was conducted in R. The mean sequencing depth of the nuclear genome after mapping quality filtering (MAPQ ≥ 25) was 1.4× for historical herbarium samples and 2.9× for modern samples.

### Genotype likelihood estimation (nuclear genome)

Genotype likelihoods were estimated for the nuclear genome with angsd v0.931 (*103*) with the options -doGLF 2, -SNP_pval 1e-6, -doMaf 3, -doGeno -1, -doPost 1, -minMapQ 25, -minQ 20, -trim 5, -minMaf 0.05, -geno_minDepth 2, -setMinDepthInd 2, -postCutoff 0.95, -remove_bads 1, -uniqueOnly 1, and -doPlink 2. Only sites with sequence data for at least half of the individuals were considered. Genotype likelihoods were first calculated on the historical herbarium dataset and the contemporary dataset separately to identify positions that were variable in both.

CallableLoci from GATK v3.7-0 (*104*) was used to estimate which parts of the nuclear reference genome were reliably mappable on the basis of mapping quality and sequencing depth distribution as described below. As the read length of historical and contemporary samples differs significantly, CallableLoci was run on historical and contemporary samples separately. The BAM files of 14 historical and 12 modern samples from both the native NA and introduced European range were merged with samtools merge v1.6 (*105*). Samples that represent the whole range and with similar sequencing depth (around 2×) were used for merging. The sequencing depth of the merged BAM files was calculated with samtools v1.6 depth with filtering for a base quality of 20 and a mapping quality of 25. The average sequencing depth was 24.3× for the merged historical BAM file and 23.0× for the merged modern BAM file. CallableLoci was run with a minimum base quality of 20, a minimum mapping quality of 25, a minimum depth of one-third of the average depth, and a maximum depth of two times the average depth. Regions with excessive sequencing depth and with low mapping quality were removed from the resulting bed files, the bed file for the modern and historic samples combined, and overlapping regions merged with bedtools merge v2.25.0.

Variant sites that were in regions with low mapping quality, as well as those within regions of excessive sequencing depth identified with CallableLoci as described above, were removed as these regions might originate from the mitochondrial or the chloroplast genome or are gene duplications and thus violate the assumption of a diploid site in the genotype likelihood estimation. The average sequencing depth in the remaining regions was 2.3× for historical herbarium samples and 4.2× for contemporary samples. In addition, only sites that were variable in both the historical herbarium dataset and the contemporary dataset, based on the genotype likelihood estimation on historic and contemporary samples separately, were extracted from the beagle file of the joint genotype likelihood estimation and used in the downstream analysis, unless otherwise stated.

The genotype likelihood estimation was once performed on the whole dataset, including other *Ambrosia* species (see table S8) to identify possible hybrids and misidentifications in the dataset. On the basis of the PCA, a total of 50 samples were removed (see table S1). In addition, 16 samples were removed because they were either first- or second-degree relatives (based on the kinship analysis described below), 23 samples were removed because of too low sequencing depth (<0.1× after filtering for a MAPQ of 25), and 4 samples were removed because of a possible sample mix-up in the laboratory. The genotype likelihood estimation was repeated on the reduced dataset containing 655 samples.

### PCA, kinship, and admixture analysis

PCangsd v.0.95 (*106*) was used to generate the covariance matrix and a kinship matrix for the whole dataset. The analysis was run until convergence to a minor allele frequency (MAF) tolerance of 0.0001. For the PCA, the R function prcomp was used with the covariance matrix. On the basis of the kinship matrix from PCangsd, first- and second-degree relatives were removed from the analyses. For each pair of related samples, the one with the highest mean sequencing depth was kept. To identify possible hybrids or misidentifications, a PCA based on the nuclear genome including different *Ambrosia* species was performed. Samples that clustered outside the main *A. artemisiifolia* group (PC1 > −1, PC2 < −0.35, and PC2 > 0 and PC1 < 0) were excluded from further analyses (fig. S8). Some outgroup samples also clustered with the main *A. artemisiifolia* cluster. This might be due to misidentifications of outgroup samples, but it is more likely the result of low sequencing depth (<0.1×) in these samples. These outgroup samples were therefore excluded in the consideration of misidentified *A. artemisiifolia* samples. In total, 47 samples (6.3% of all samples) were excluded: 34 historical European (13%), 10 historical NA (8.5%), and 3 contemporary NA (1.5%) samples (table S1). The PCA was repeated including only the final set of samples. For the admixture and PCA analyses, sites in with linkage disequilibrium (LD) ≥ 0.5 were removed. LD was estimated using Plink v1.90 (*107*) with a window size of 50 and a step size of 5. In addition, only sites variable in both historical and modern samples were used. A total of 1,094,260 sites were used for the PCA and admixture analysis after filtering for MAF of 0.05. NGSadmix (*108*) was run on the reduced dataset with up to 15 ancestral populations (*K*). Ten independent runs with different seeds were performed for each *K* value. CLUMPAK Distruct (*109*) was used to align the output files for different *K* values. The run with the highest likelihood for each *K* was used for plotting. *K* = 9 was chosen for the main manuscript as it was the most likely according to the MAP test in PCangsd and showed a peak with the Δ*K* method (*110*). Plots for the other *K* values can be found in the Supplementary Materials (figs. S9 to S22).

In the native range, admixture results correlate with geographic sample location. As we sampled from a continuous range, the admixture results were used to inform the boundaries of larger populations (East, Mideast, West, and South). To be able to assign European samples to these native populations, we used admixture proportions for *K* = 9 to group samples. Samples that had at least 55% ancestry of the dominant genetic cluster in the South, East, or West of NA, respectively, were assigned to the South, East, or West population. For the remaining samples from NA, all were confined to the geographic area between these three regions. These samples were not assigned to one dominant cluster but shared in common a combined ancestry of at least 70% of the East, West, and South clusters, as well as a fourth unnamed cluster. Given the similarity in their genetic composition and their geographic proximity, we termed this set of samples the Mideast population. Samples from the introduced European range were assigned to these populations in the admixture analysis but were kept as a separate population for all between-population analysis. See fig. S6 for the population assignment in the native range.

### Ancestral state estimation

To generate the ancestral state for common ragweed, shotgun sequencing reads of two closely related (*111*) species were used (*Ambrosia chamissonis* and *Ambrosia carduacea*) and mapped against the *A. artemisiifolia* reference genome. The sequencing depth after MAPQ 25 filtering for both samples is 3.5×. To generate the ancestral state fasta file, angsd v0.931 (*103*) -doFasta 2 was used with a minimum base quality of 20, minimum mapping quality of 25, and the options -remove_bads 1, -uniqueOnly 1, and -explode 1.

### Heterozygosity, *F*_ST_, and *N*_e_ estimation

For each sample, heterozygosity was estimated over the whole genome. First, the site allele frequency (SAF) was estimated with angsd v0.931 (*103*) using -dosaf 1, -minMapQ 25, -minQ 20, -remove_bads 1, -uniqueOnly 1, and -trim 5. The site frequency spectrum (SFS) was polarized using the ancestral state estimated from *A. chamissonis* and *A. carduacea*. To test whether differences in heterozygosity are due to differences in sequencing depth between modern and historical samples, all samples with sequencing depth above 1×, 0.75×, 0.5×, and 0.25×, respectively, after filtering for mapping quality 25 were downsampled to ~1×, ~0.75×, ~0.5×, and ~0.25× sequencing depth with the -downsample option during SAF estimation. The heterozygosity estimates including all reads for each sample and downsampled to 1×, 0.75×, and 0.5× sequencing depth are strongly correlated (fig. S23). The correlation when downsampling to 0.2× is lower, with *R*^2^ < 0.9. Thus, samples with sequencing depths below 0.5× after MAPQ 25 filtering were removed from the analysis to avoid bias due to low sequencing depth. To test whether there are significant differences in heterozygosity between populations, a Mann-Whitney *U* test was performed in R v3.4.4.

For the *F*_ST_ estimation, samples were grouped by populations (East, West, South, Mideast, and Europe; Fig. 1, C and D) and by time (historic and modern). Population assignment was based on admixture results for *K* = 9 (see admixture analysis). Four samples from three sampling locations were assigned to either the Mideast or East population but were geographically not located in that population (fig. S6). These samples were excluded from their respective population for all population-based analysis. Samples that are first- or second-degree relatives and those that are misidentifications or possible hybrids based on the PCA analysis including outgroups from the genus *Ambrosia* were removed. First, the SAF was estimated with angsd using the command line options -minMapQ 25, -minQ 20, -remove_bads 1, -uniqueOnly 1, and -trim 5. The SFS was polarized using the ancestral state estimated from *A. chamissonis* and *A. carduacea*.

To estimate the effective population size *N*_e_, thetas were calculated with the angsd realSFS saf2theta tool for each population, polarized using the ancestral state estimated from *A. chamissonis* and *A. carduacea*. The mean of the Watterson estimator across the genome was used to calculate *N*_e_, and the 95% confidence interval of the mean was used to calculate the error of *N*_e_. A mutation rate of 1 × 10^−8^ substitutions per site per generation [estimate from the closely related species *Helianthus annuus* (*112*)] and a generation time of 1 year were used, as *A. artemisiifolia* is an annual plant.

### Definition of spatial groups

Samples within a radius of 100 km were clustered into spatial groups using the R package geosphere, and the centroid of the radius was used as the location of the spatial groups. If a spatial group contained at least four samples, then we selected that group as a “spatial group” in which changes in the genetic structure potentially could be directly observed. For some spatial groups, at least four samples were available from before 1900 and from between 1900 and 1940. For those spatial groups, we split the historical spatial group into an older (<1900) and a younger (1900–1940) spatial group for a higher temporal resolution. To create modern geographic groups, we selected from available modern populations that were collected for previous studies or collected new samples in close proximity to the historical spatial groups. For one spatial group (Hamburg), no modern samples could be found because of successful eradication of the invasive plant. In NA, we chose some additional modern locations where less than four historical samples were available. We used these locations to gain a better resolution.

Pairwise *F*_ST_ between spatial groups was calculated with realSFS from angsd (*103*, *113*). The SFS was polarized using the ancestral state. All pairwise *F*_ST_ estimates can be found in table S9. Geographic distance between the centroid of the spatial groups was calculated with the distm function in R. An MDS analysis was performed on the *F*_ST_ distance matrix in R with maxit=5000. For the IBD analysis, a Mantel test was performed using the gl.ibd function of the R package dartR in R v4.1.1 with 999 permutations. IBD was tested within historic North America, modern North America, historic Europe, and modern Europe.

To test for introgression of *A. trifida* and *A. psilostachya*, the multipopulation *D*-statistic (Abbababa2) within angsd (*103*, *114*) was used. *A. carduacea* was used as an outgroup as its distribution [Western North America (*115*)] does not overlap with that of *A. artemisiifolia*, and thus, introgression from this species is unlikely. *D*-statistics of the form (E, H2, *A. trifida*, *A. carduacea*), (N, H2, *A. trifida*, *A. carduacea*), (E, H2, *A. psilostachya*, *A. carduacea*), and (N, H2, *A. psilostachya*, *A. carduacea*) were considered, where N is a spatial group from North America and E a spatial group from Europe. All possible comparisons with E, N, and H2 being from the same time period and H2 being a NA spatial group, excluding those that themselves showed signs of introgression (modern and historical StLouis and modern and historical Ames for *A. trifida*; modern and historical FLSouth, modern FLNorth, and historical Ames for *A. psilostachya*) were calculated, and the mean value of these was used for plotting. For each spatial group, the admixture diversity score was calculated as in (*116*) based on *K* = 9 from the admixture analysis including all samples.

### Pairwise sequentially Markovian coalescent

To infer the effective population size change history of common ragweed, we used the PSMC model (*117*). The sample used for PSMC was QC-2-30 with an average sequencing depth of 10×. To generate the diploid consensus sequence, we applied the sequencing depth filter of >^1^/_3_ and <2× of the average sequencing depth and filtered reads with a minimum mapping quality of 25 and a minimum base quality of 25 using samtools (*105*). Only scaffolds longer than 100 kbp were used for PSMC. PSMC was then applied to infer the density of the time to the most recent common ancestor between the two haploids across the genome. Demographic history was calculated assuming a generation time of 1 year (*35*) and a mutation rate of 1.0 × 10^−8^ substitutions per site per generation (*112*). The robustness of the inference was estimated with 100 bootstraps (fig. S4).

### Selection scanning

To identify genes putatively under selection in Europe, *F*_ST_ was estimated in nonoverlapping sliding windows with a window size of 10 kbp between historical and modern Europe, as well as between modern Europe and modern North America. As the South population did not seem to have contributed to the European invasion, it was excluded from the NA population for this analysis. The SFS for each population was estimated with angsd (*103*, *113*), excluding sites with excessive coverage or low MAPQ and only including sites that were covered in at least two-thirds of the samples, with an MAPQ of ≥25 and a minimum base quality of 20. Reads were excluded if they had multiple best hits or if they were tagged as not primary alignment, failure, or duplicate reads. The SFS was polarized using the ancestral state (see ancestral state estimation section). realSFS from angsd (*103*, *113*) was used to generate the two-dimensional SFS between population pairs and generate *F*_ST_ in sliding windows. Only windows with at least 100 SNPs were considered. *F*_ST_ values were *z*-transformed, and a cutoff of *z* > 6 was used for outlier windows. The polarized site frequency spectrum was used to generate neutrality tests in the same windows as *F*_ST_ with the thetaStat tool in angsd (*118*). Fay and Wu’s *H* (*52*) was extracted for *F*_ST_ outlier and nonoutlier windows, and statistical significance was estimated with a Wilcoxon rank sum test in R.

GO enrichment was assessed using GO terms from *A. thaliana* TAIR 10 (*89*) BLAST results. To identify GO terms enriched among candidate lists, the topGO package in R was used with Fisher’s exact test, the weight01 algorithm, and a *P* < 0.05 threshold to assess significance. Only GO terms describing biological processes were considered. In addition, *F*_ST_ between individual SNPs was *z*-transformed, and outliers were defined as those with *z* > 50. The annotation was used to examine whether SNPs are located within gene regions. Functional annotation was obtained from *A. thaliana* TAIR 10 (*89*) BLAST results. In addition, annotations were cross-referenced with 306 *A. thaliana* genes known to be involved in flowering time (*51*). A total of 566 predicted *A. artemisiifolia* genes were matched to this dataset, representing 191 unique *A. thaliana* flowering time genes.

### Metagenomic community classification

Reads that did not map against the *A. artemisiifolia* reference genome were used to analyze the leaf metagenome. Unmapped reads were extracted from the resulting BAM files using samtools v1.9 (*105*) and converted into fastq files with PicardTools v2.21.2 SamToFastq (http://broadinstitute.github.io/picard). Samples that were grown from seeds in the greenhouse were excluded from the analysis (see table S1).

For the metagenomic classification, PCR duplicates were removed with clumpify from the BBMap package (sourceforge.net/projects/bbmap/) using the default parameters. The unmapped reads were then classified with kraken2 (*119*) using the NCBI_non-redundant_ntdb database (downloaded August 2020) in paired-end mode for the paired data and in single-end mode for overlapping reads that were collapsed into a single read during adapter removal. As this study focuses on microbes, reads assigned to Metazoa or Viridiplantae were removed with the extract_kraken_reads.py script from KrakenTools (github.com/jenniferlu717/KrakenTools/). The two kraken reports for paired and collapsed reads for each sample were combined into one report using the combine_kreports.py script from KrakenTools. Kraken-biom v1.0.1 (github.com/smdabdoub/kraken-biom/) was used to combine the results from all samples for subsequent analysis in R v4.0.3.

Thereafter, several filters were applied to the dataset to exclude particular taxa from further analysis. Only taxonomic identifications at the species level were used. Species with a relative abundance below 0.05% were removed under the assumption that they are likely false positives. The package decontam (*120*) was used to identify taxa that are probable laboratory contaminants based on the extraction blanks prepared alongside the historical and modern samples using the “prevalence” method with a threshold of 0.4. Taxa identified as contamination, uncultured taxa, and cloning vectors were removed. As we saw a large difference between historical herbarium and contemporary samples that we could not rule out as herbarium contamination, the analysis was restricted to prokaryotic plant pathogens identified by the FAPROTAX database (*59*) as these are less likely to be herbarium contamination. Abundance data were transformed into presence/absence, and significant differences were evaluated by using a two-sample *t* test.

## Notes

View/request a protocol for this paper from *Bio-protocol*.
