# Supplementary material for: Uncovering the genomic basis of an extraordinary plant invasion
Source: Sci Adv. 2022 Aug 24;8(34):eabo5115. doi: 10.1126/sciadv.abo5115 (PMC9401624; doi:10.1126/sciadv.abo5115)
Supplement: Supplementary file 1 — Supplementary Text Figs. S1 to S23 Table S4 [file sciadv.abo5115_sm.pdf]

Science Advances
AAAS

Supplementary Materials for

Uncovering the genomic basis of an extraordinary plant invasion

Vanessa C. Bieker *et al.*

Corresponding author: Vanessa C. Bieker, vanessa.bieker@gmx.de; Michael D. Martin, mike.martin@ntnu.no

Sci. Adv. **8**, eabo5115 (2022)
DOI: 10.1126/sciadv.abo5115

The PDF file includes:

Supplementary Text
Figs. S1 to S23
Tables S4
Legends for table S1 to S3, and S5 to S9

Other Supplementary Material for this manuscript includes the following:

Table S1 to S3, and S5 to S9

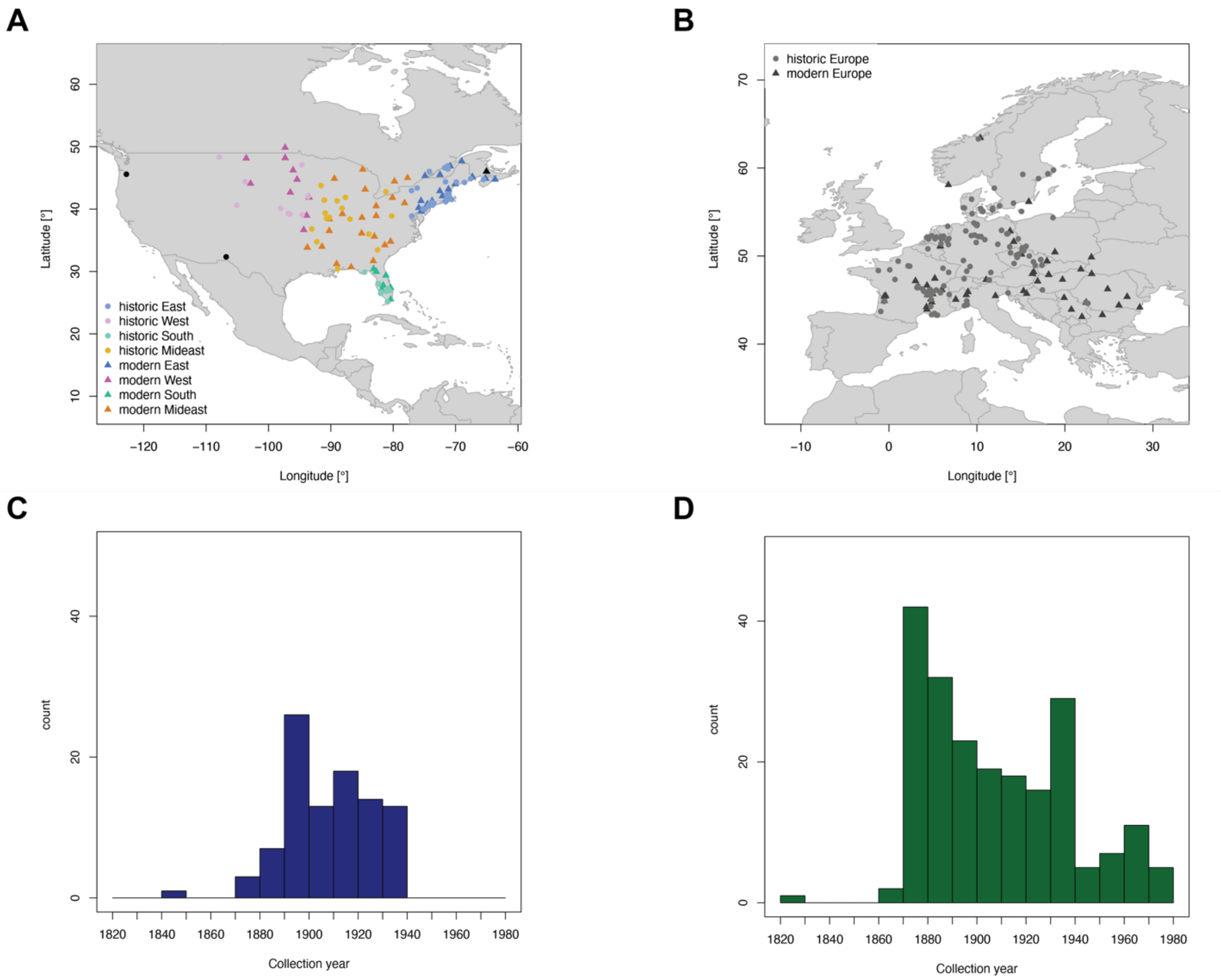

Fig. S1.

Sampling locations, population assignment and collection year. (**A**) North America. Light blue circles: historical East, dark blue triangles: modern East, light pink circles: historical West, dark pink triangles: modern West, light turquoise circles: historical South, dark turquoise triangles: modern South, light orange circles: historical Mideast, dark orange triangles: modern Mideas, black circles: historical samples that geographically do not group with their population and were thus excluded from population comparisons, black triangles: modern samples that geographically do not group with their population and were thus excluded from population comparisons. (**B**) Europe. grey circles: historical samples, black triangles: modern samples. (**C**) Histogram of the collection years of North American historical herbarium samples. (**D**) Histogram of the collection years of European historical herbarium samples.

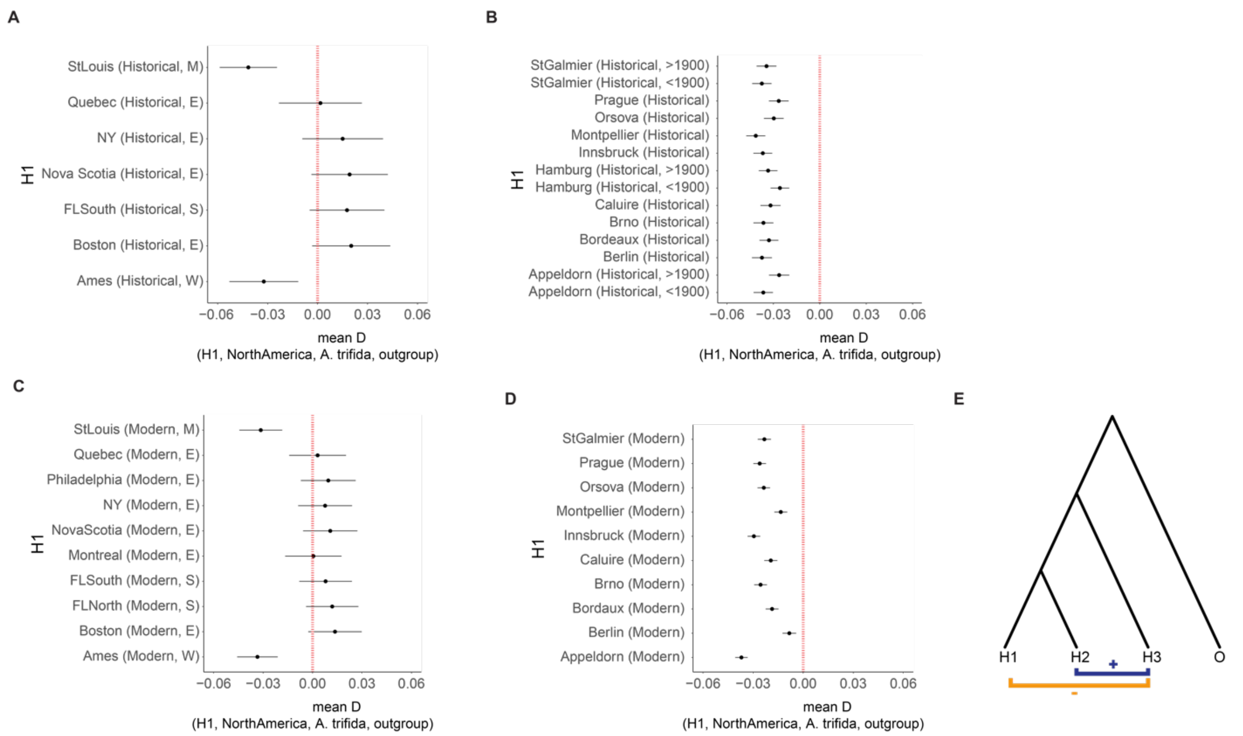

Fig. S2.

D statistics of the form (H1, North American population, *Ambrosia trifida*, *Ambrosia carduacea*). Plotted are the mean D values for all possible comparisons where H1 and North American population are not the same population. Error bars indicate one standard deviation from the mean. For North American spatial groups, population assignment is given in parentheses with M: Mideast, E: East, S: South, W: West. (**A**) H: Historical North American populations. (**B**) H1: Historical European populations (**C**) H1: Modern North American populations. (**D**) H1: Modern European populations. (**E**) Schematic tree showing the direction of gene flow for positive (blue) and negative (orange) D values.

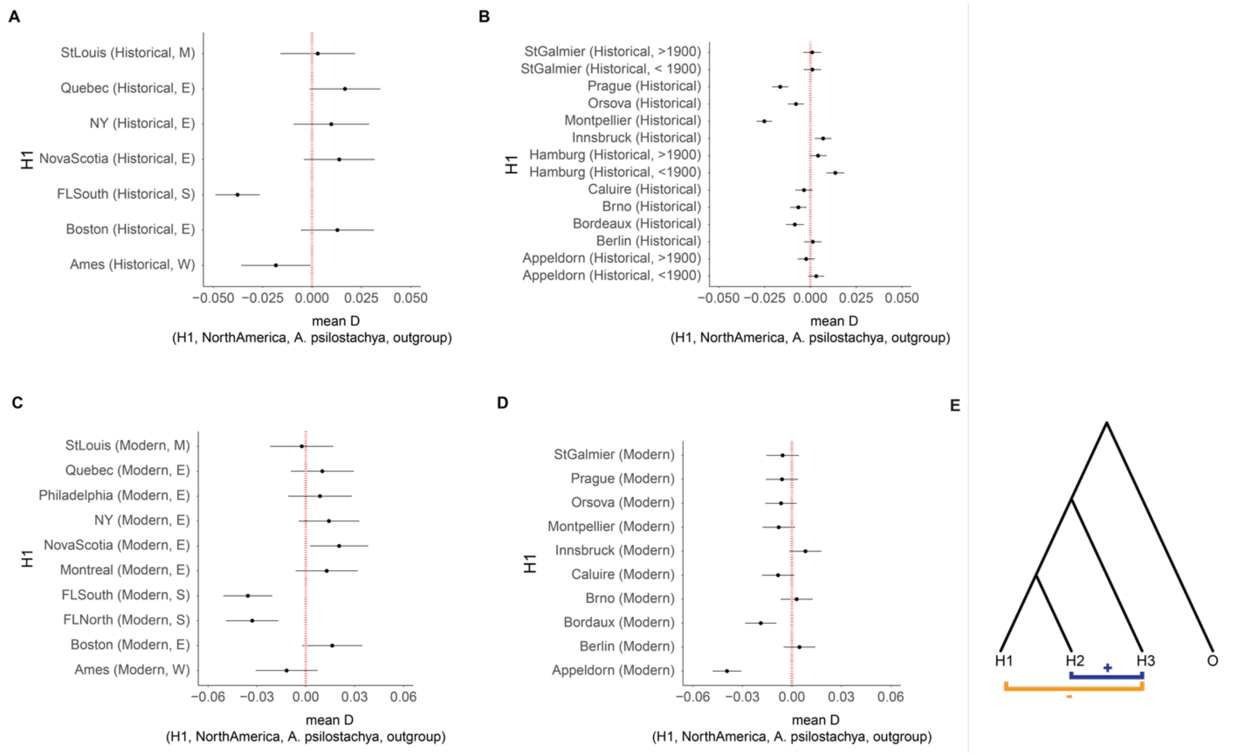

Fig. S3.

D statistics of the form (H1, North American population, *Ambrosia psilostachya*, *Ambrosia carduacea*). Plotted are the mean D values for all possible comparisons where H1 and North American population are not the same population. Error bars indicate one standard deviation from the mean. For North American spatial groups, population assignment is given in parentheses with M: Mideast, E: East, S: South, W: West. (**A**) H: Historical North American populations. (**B**) H1: Historical European populations (**C**) H: Modern North American populations. (**D**) H1: Modern European populations. (**E**) Schematic tree showing the direction of gene flow for positive (blue) and negative (orange) D values.

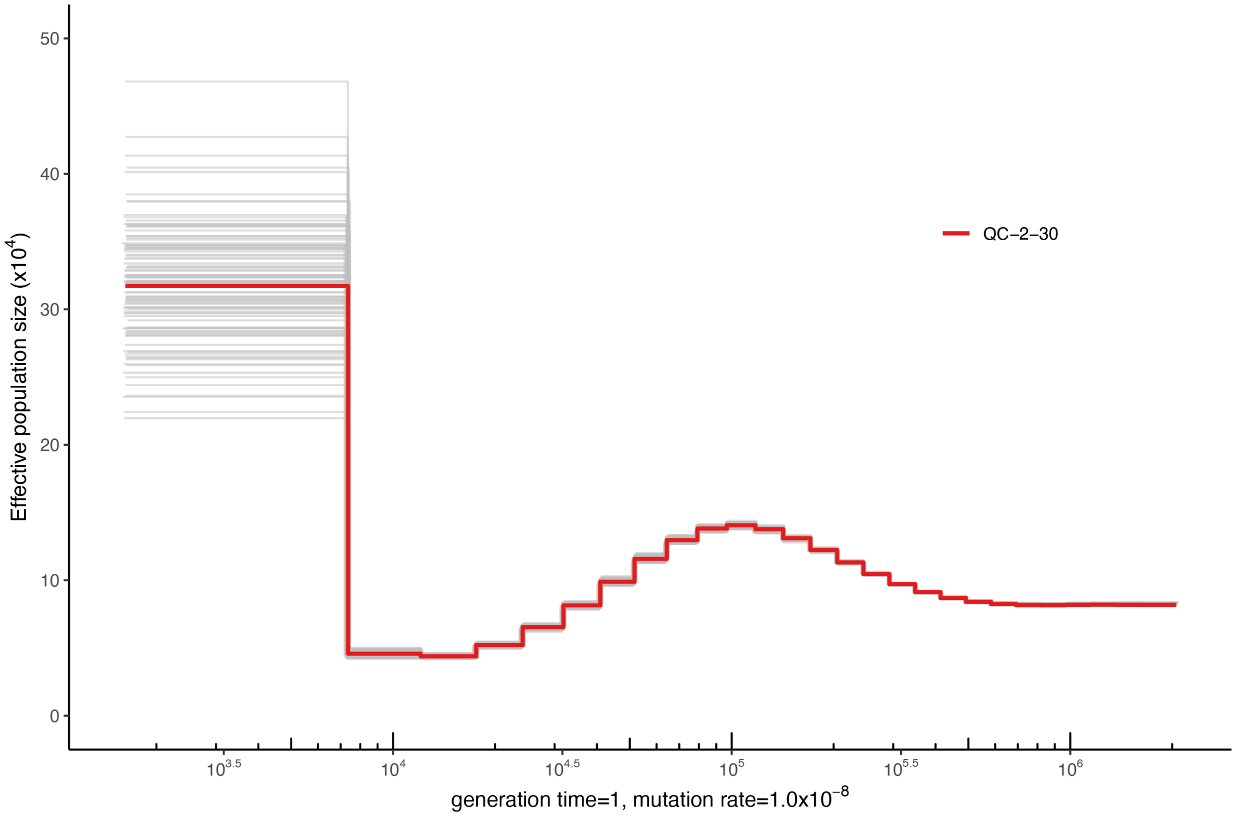

Fig. S4.

Demographic history of ragweed inferred by PSMC. We calibrated the effective population size assuming a mutation rate of 1.0 10^{-8} per site per generation and the generation time of ragweed as 1 year. The grey lines represent 100 bootstraps. N. American individual QC-2-30 was used for demographic inference.

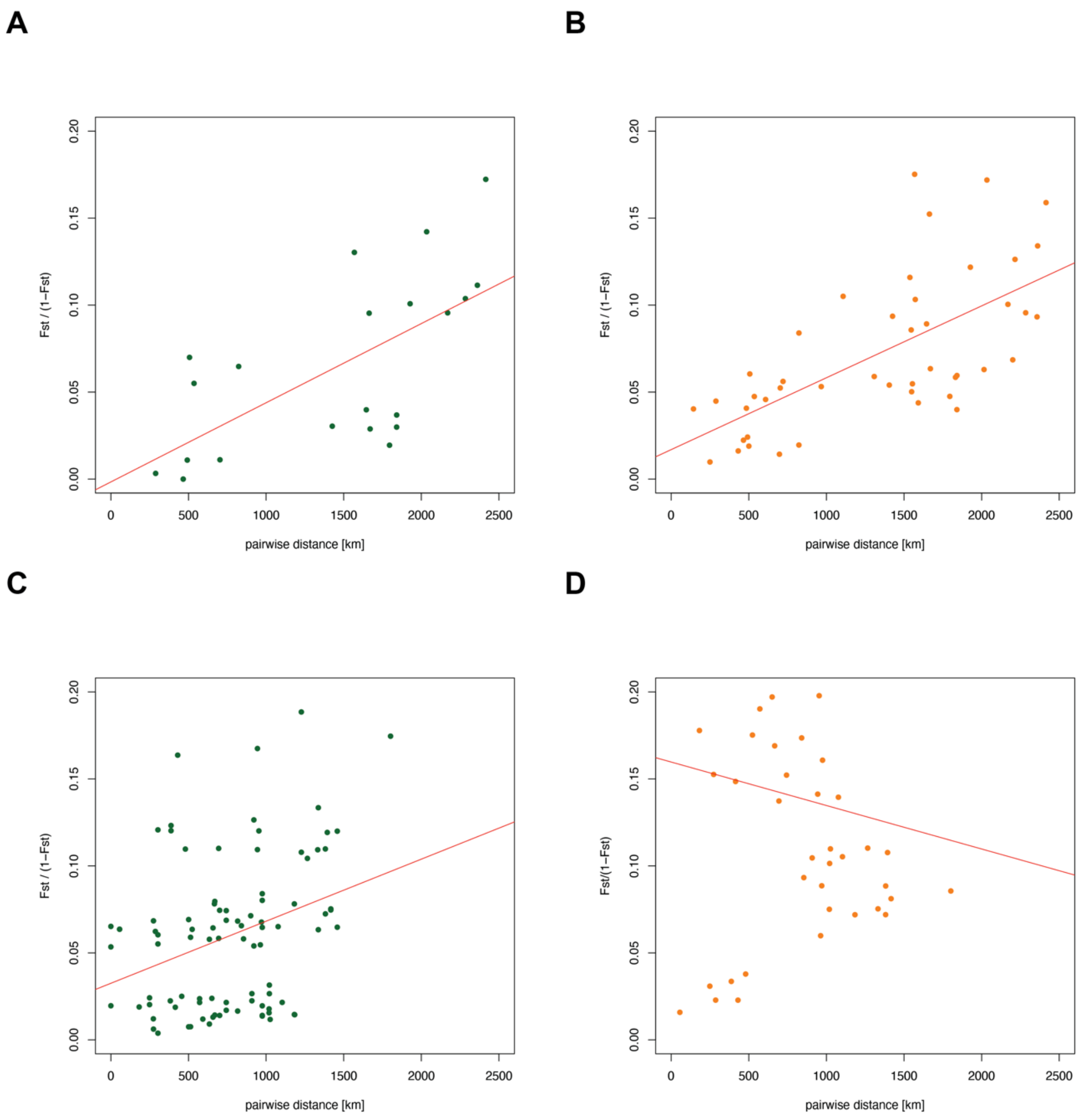

Fig. S5.

Isolation by distance in the native (top) and introduced (bottom) range. The red line represents the linear regression line. (**A**) historical North America (Mantel statistic r: 0.6462, significance: 0.004), (**B**) modern North America (Mantel statistic r: 0.6578, significance: 0.001), (**C**) historical Europe (Mantel statistic r: 0.3244, significance: 0.051), (**D**) modern Europe (Mantel statistic r: -0.1234, significance: 0.696).

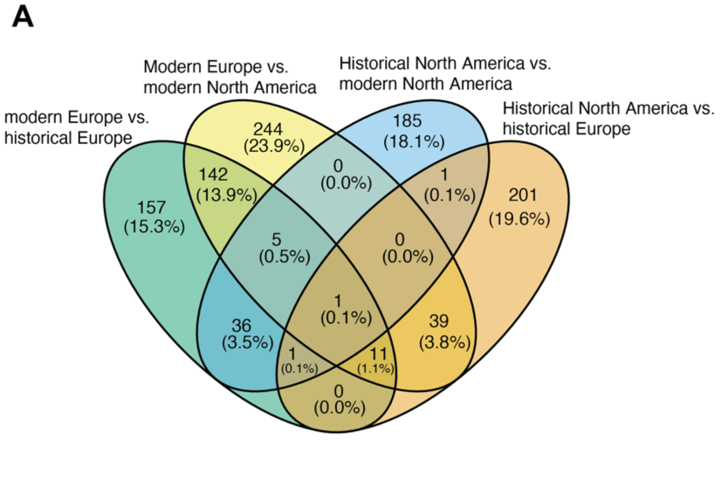

Fig. S6.

Venn diagram of overlapping F_{ST} outlier windows between the comparisons. (**A**) Overlapping windows between all four comparisons. Green: modern Europe vs. historical Europe, yellow: modern Europe vs. modern North America, blue: historical North America vs. modern North America, orange: historical North America vs. modern North America. (**B**) Overlapping windows between modern Europe vs. historical Europe (green) and Historical North America vs. modern North America (blue). (**C**) Overlapping windows between modern Europe vs. historical Europe and modern Europe (green) and modern North America (yellow).

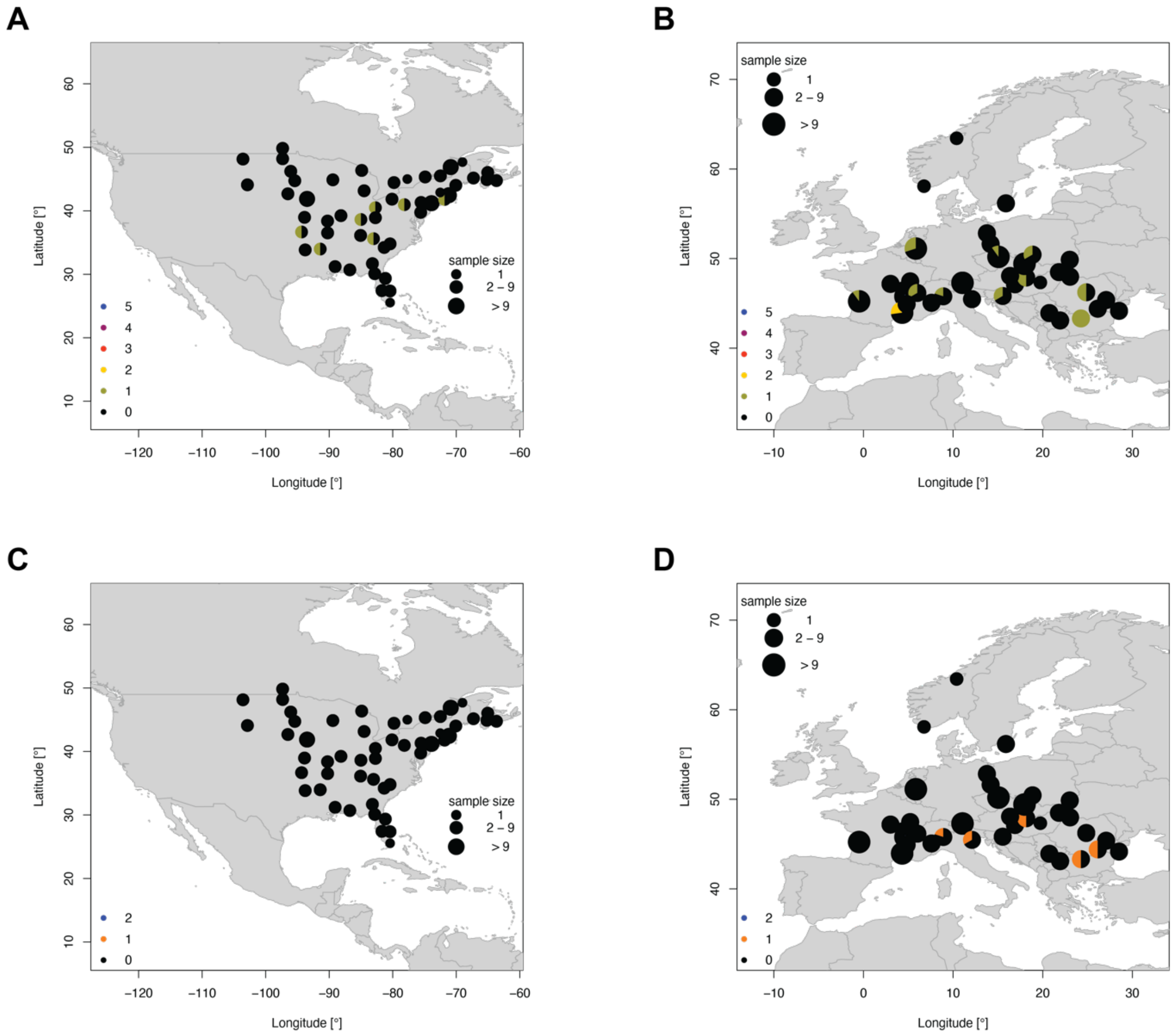

Fig. S7.

Pathogen prevalence in North America and Europe. (**A**) Prevalence of *Dickeya* spp. in North America. (**B**) Prevalence of *Dickeya* spp. in Europe (**C**) Prevalence of *Brennaria* spp. species in North America (**D**) Prevalence of *Brennaria* spp. species in Europe. Samples within 100 km were grouped together. The pie chart indicates the fraction of samples in which *Brennaria* spp. or *Dickeya* spp. are present with black indicating no *Brennaria*/*Dickeya* species identified. The color indicates how many different species were identified at a location.

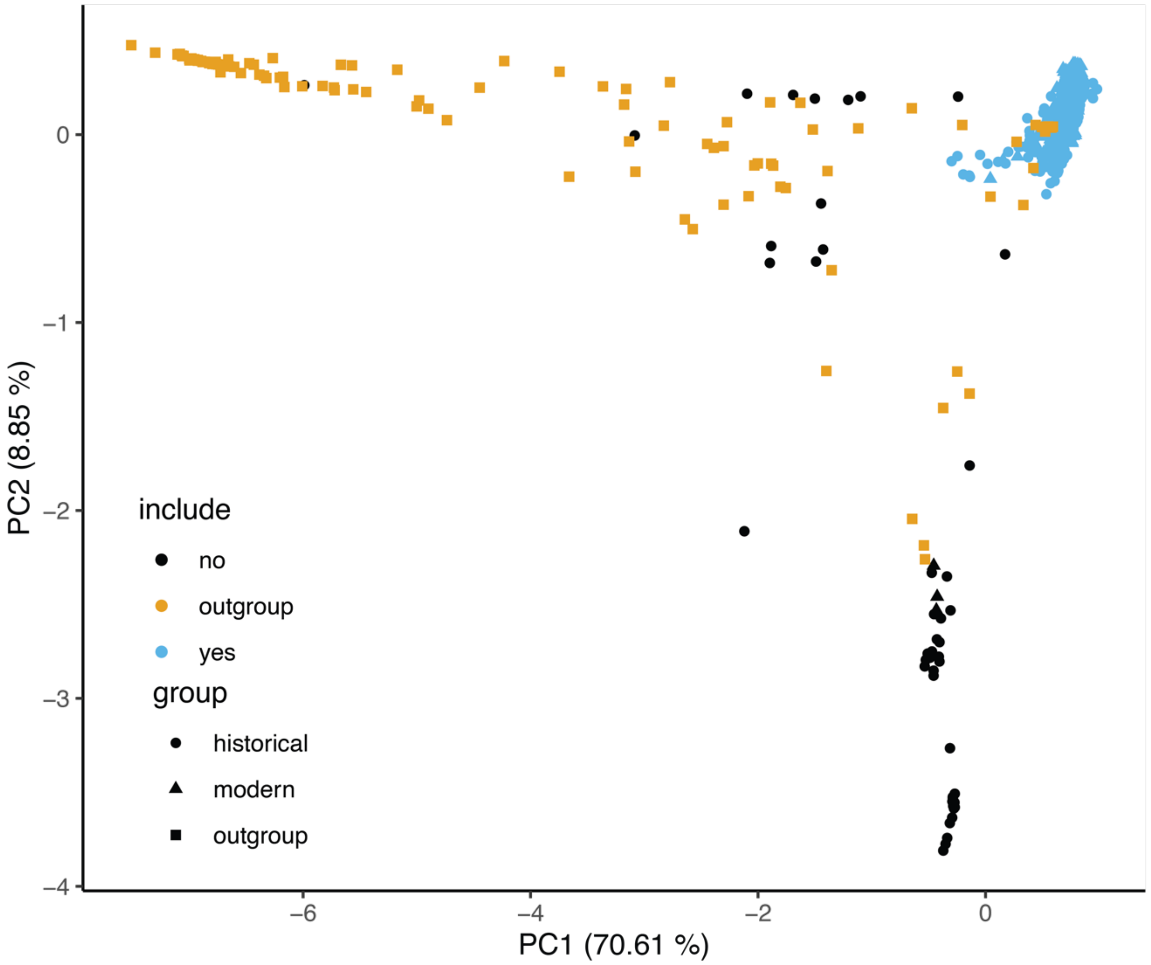

Fig. S8.

PCA including outgroup samples (other *Ambrosia* species). Orange squares: outgroup samples, black circles: historical samples that were excluded based on this PCA, blue circles: historical samples that were included in the final analysis, black triangles: modern samples that were excluded based on this PCA, blue triangles: modern samples that were included in the final analysis.

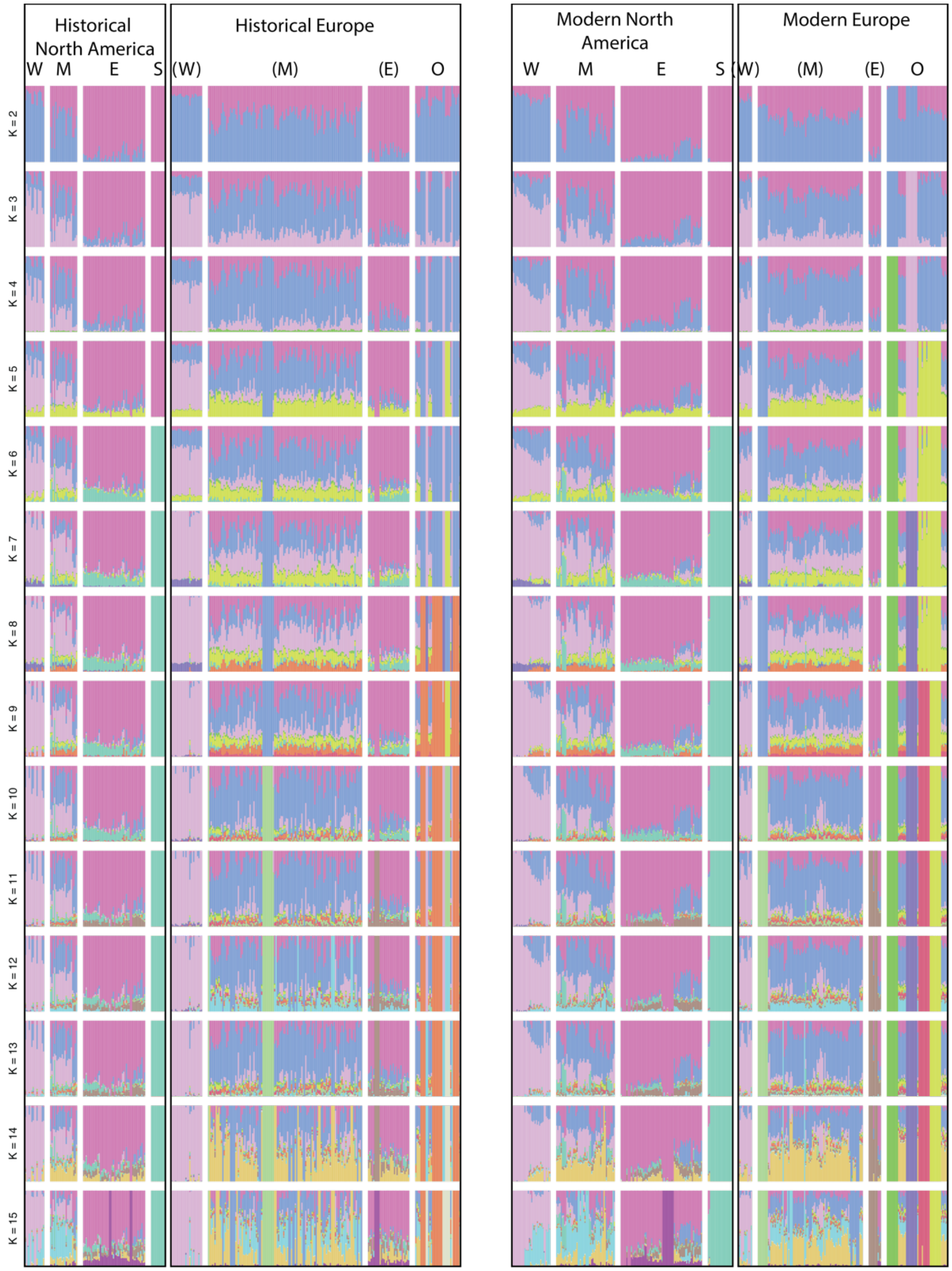

Fig. S9.

Population structure obtained from NGSadmix for K=2-15. NGSadmix was run with all samples and the run with the highest likelihood is plotted. Samples are grouped based on their assignment to a North American population (W: West, M: Mideast, E: East, S: South, O: samples that could not be assigned to a North American population).

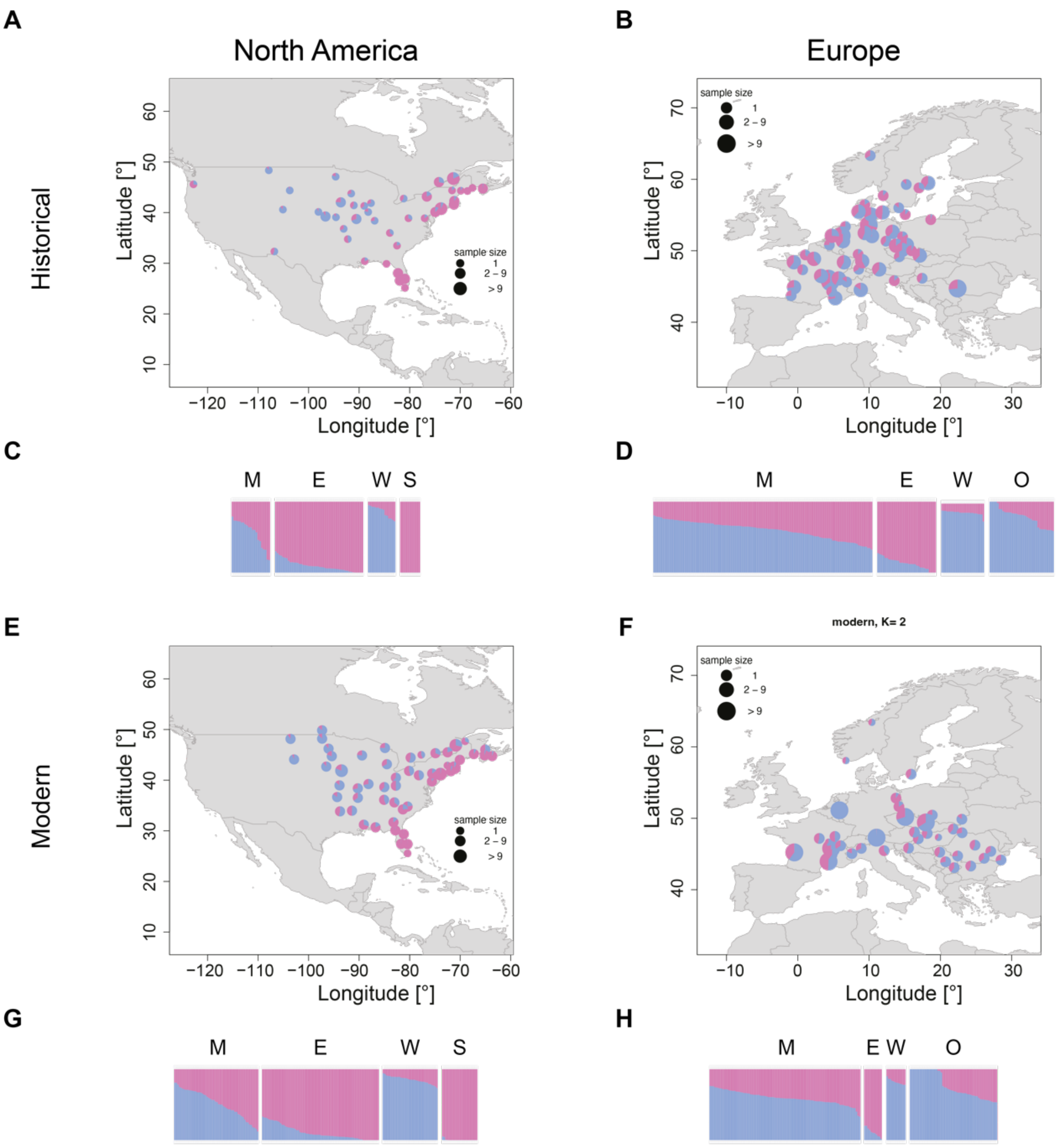

Fig. S10.

Population structure obtained from NGSadmix for K=2. The NGSadmix run of a joint analysis including all samples with the highest likelihood was used for plotting. The same color scheme was used across all panels. (**A, B, E, F**) Admixture maps. Samples within 100 km were grouped together and the average ancestry across those groups was plotted. If samples were grouped together, ancestry values were plotted at the centroid of the group. (**C, D, G, H**) Admixture barplots. Each bar represents one individual. Samples are grouped based on their assignment to a genetic cluster (based on K=9): M: Mideast, E: East, W: West, S: South, O: other. (A) Historical North America. (B) Historical Europe. (C) Historical North America. (D) Historical Europe. (E) Modern North America. (F) Modern Europe. (G) Modern North America. (H) Modern Europe.

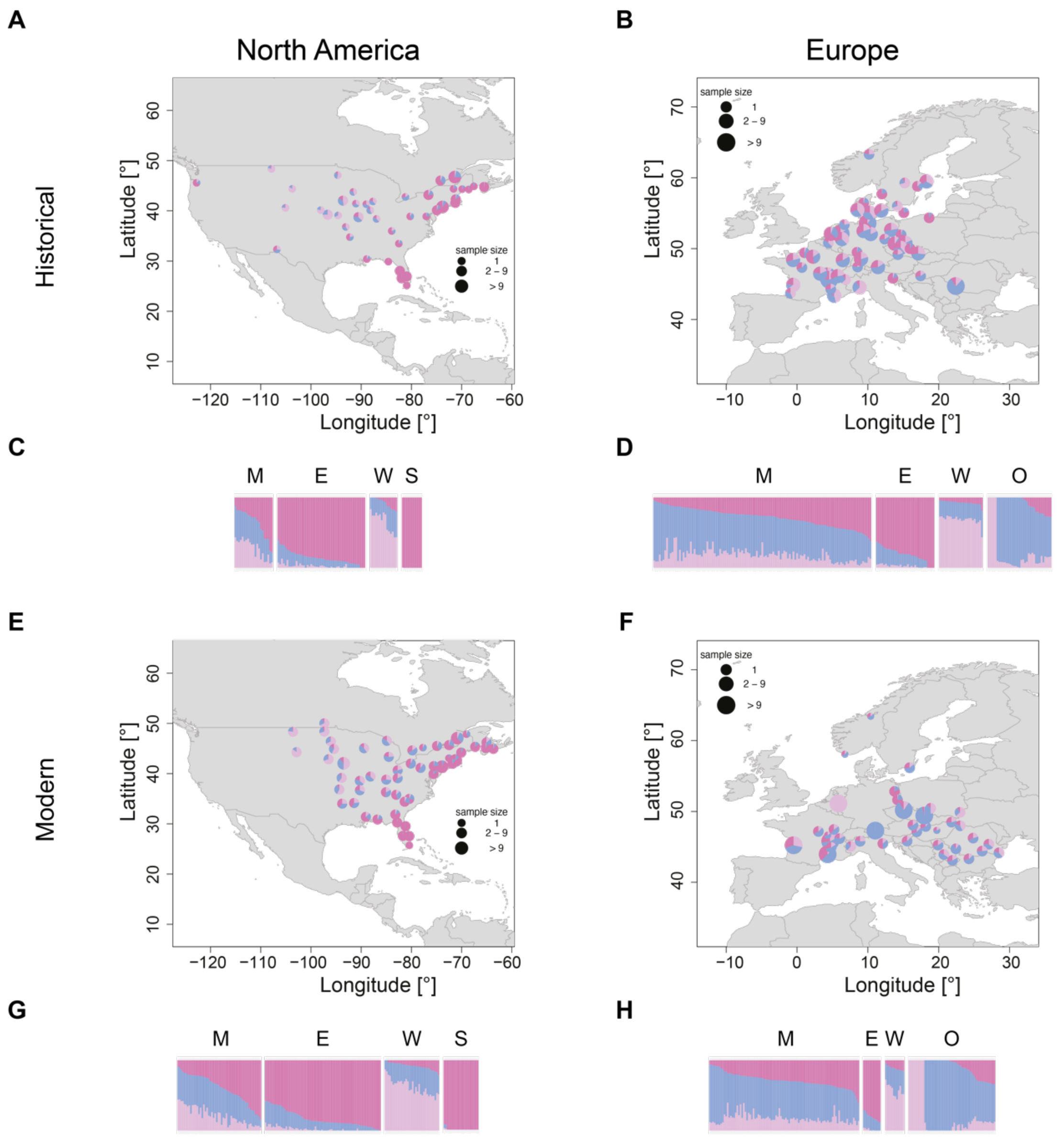

Fig. S11.

Population structure obtained from NGSadmix for K=3. The NGSadmix run of a joint analysis including all samples with the highest likelihood was used for plotting. The same color scheme was used across all panels. (**A, B, E, F**) Admixture maps. Samples within 100 km were grouped together and the average ancestry across those groups was plotted. If samples were grouped together, ancestry values were plotted at the centroid of the group. (**C, D, G, H**) Admixture barplots. Each bar represents one individual. Samples are grouped based on their assignment to a genetic cluster (based on *K*=9): M: Mideast, E: East, W: West, S: South, O: other. (A) Historical North America. (B) Historical Europe. (C) Historical North America. (D) Historical Europe. (E) Modern North America. (F) Modern Europe. (G) Modern North America. (H) Modern Europe.

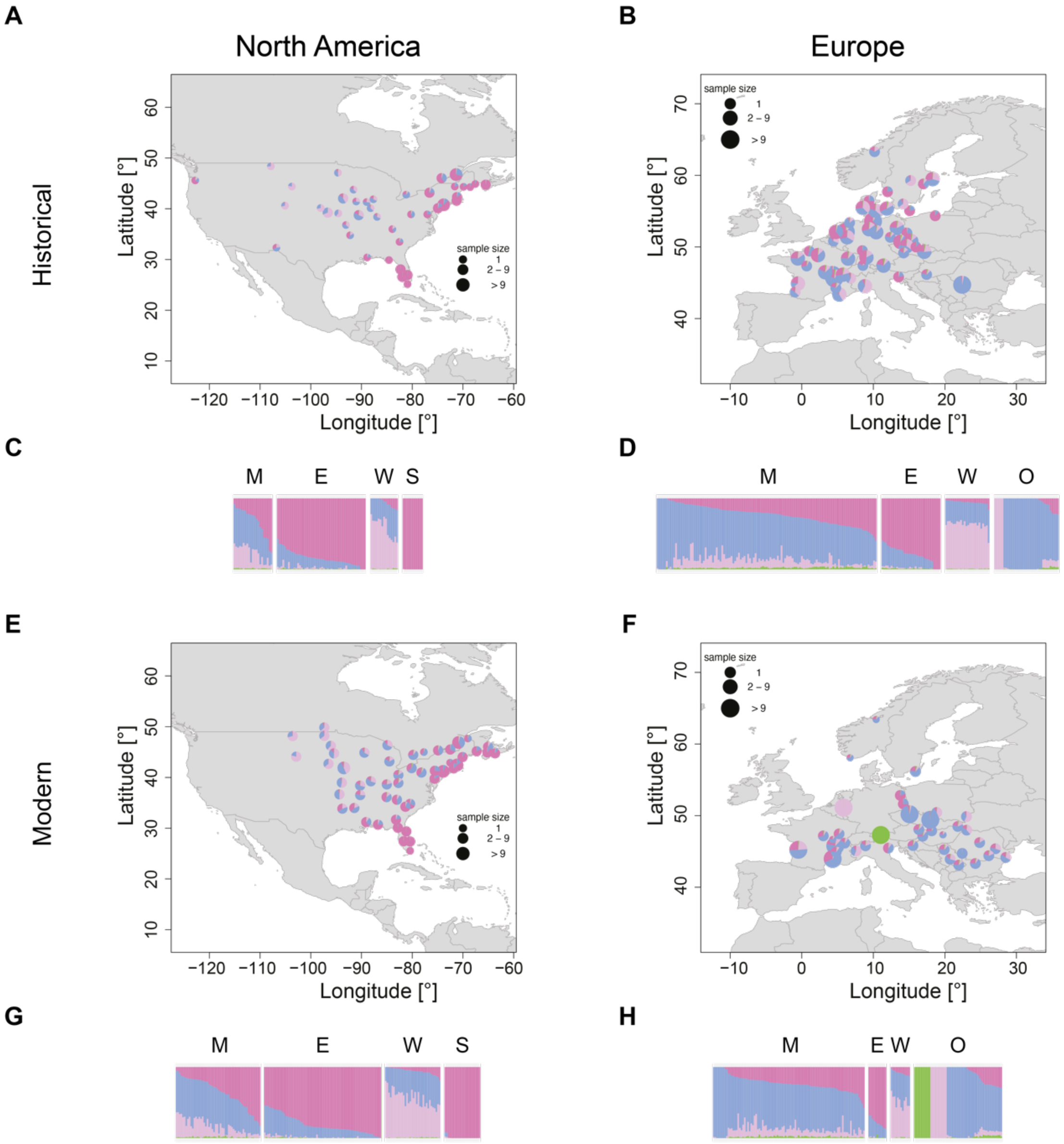

Fig. S12.

Population structure obtained from NGSadmix for K=4. The NGSadmix run of a joint analysis including all samples with the highest likelihood was used for plotting. The same color scheme was used across all panels. (**A, B, E, F**) Admixture maps. Samples within 100 km were grouped together and the average ancestry across those groups was plotted. If samples were grouped together, ancestry values were plotted at the centroid of the group. (**C, D, G, H**) Admixture barplots. Each bar represents one individual. Samples are grouped based on their assignment to a genetic cluster (based on K=9): M: Mideast, E: East, W: West, S: South, O: other. (A) Historical North America. (B) Historical Europe. (C) Historical North America. (D) Historical Europe. (E) Modern North America. (F) Modern Europe. (G) Modern North America. (H) Modern Europe.

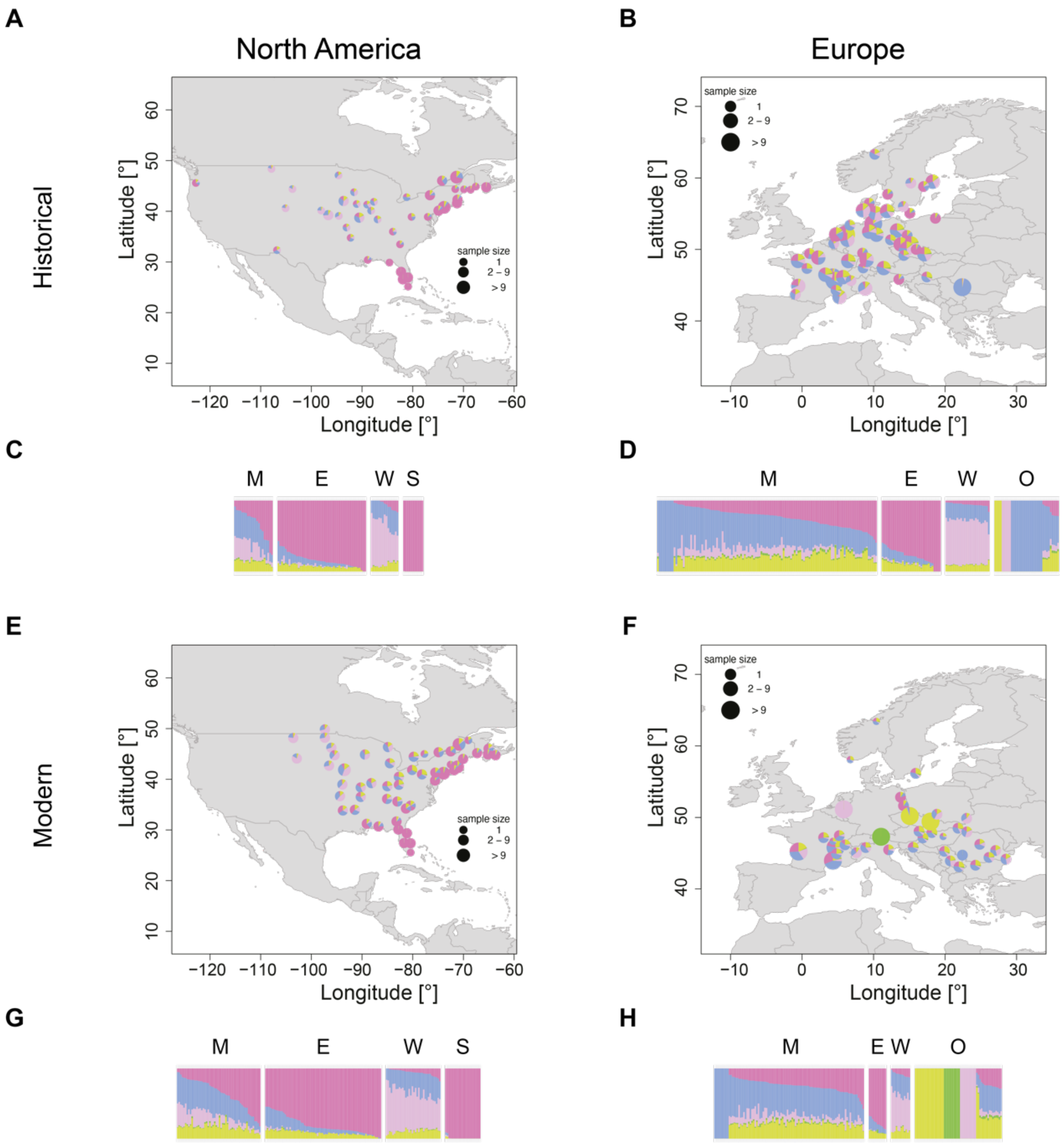

Fig. S13.

Population structure obtained from NGSadmix for K=5. The NGSadmix run of a joint analysis including all samples with the highest likelihood was used for plotting. The same color scheme was used across all panels. (**A, B, E, F**) Admixture maps. Samples within 100 km were grouped together and the average ancestry across those groups was plotted. If samples were grouped together, ancestry values were plotted at the centroid of the group. (**C, D, G, H**) Admixture barplots. Each bar represents one individual. Samples are grouped based on their assignment to a genetic cluster (based on *K*=9): M: Mideast, E: East, W: West, S: South, O: other. (A) Historical North America. (B) Historical Europe. (C) Historical North America. (D) Historical Europe. (E) Modern North America. (F) Modern Europe. (G) Modern North America. (H) Modern Europe.

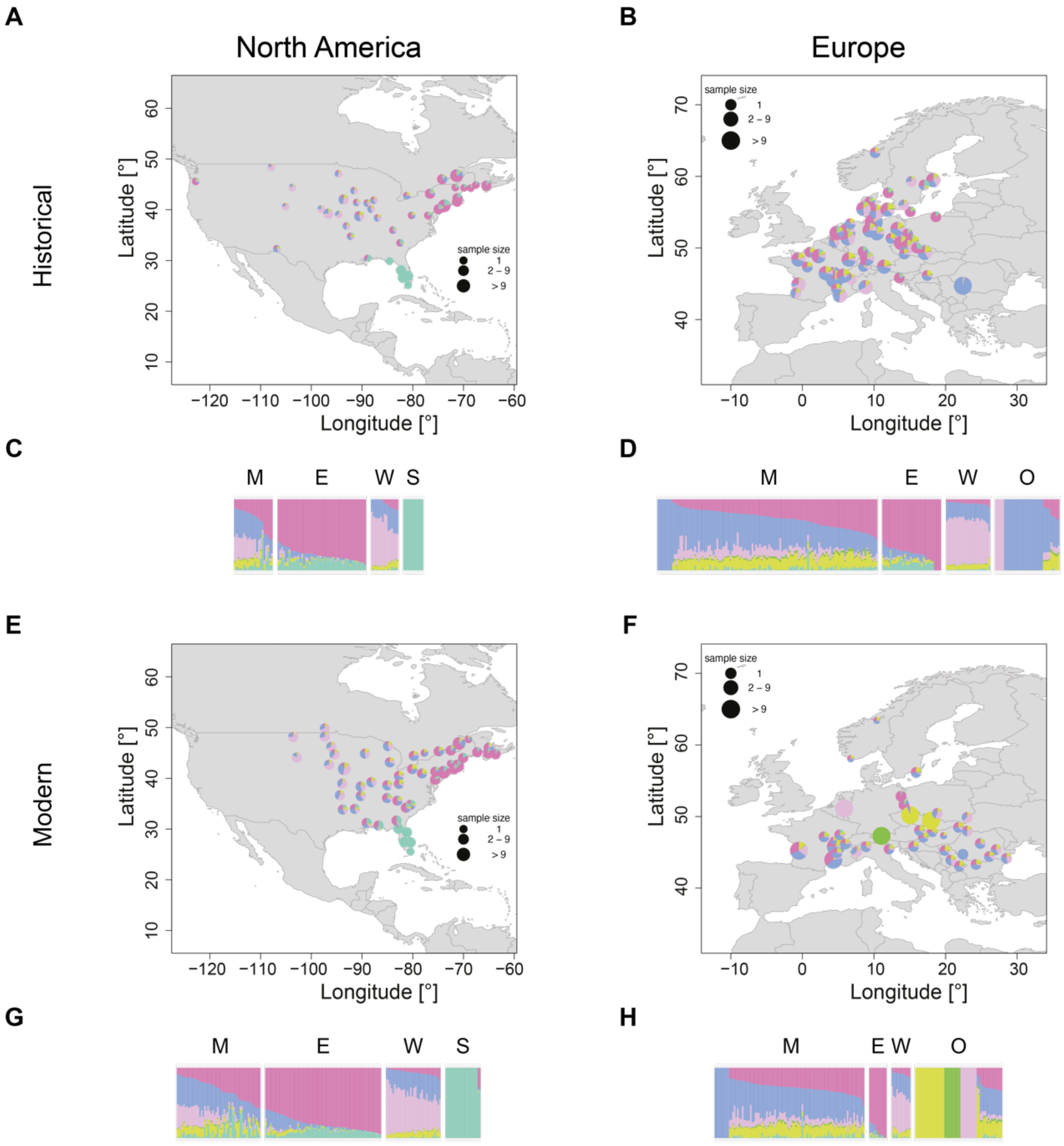

Fig. S14.

Population structure obtained from NGSadmix for K=6. The NGSadmix run of a joint analysis including all samples with the highest likelihood was used for plotting. The same color scheme was used across all panels. (**A, B, E, F**) Admixture maps. Samples within 100 km were grouped together and the average ancestry across those groups was plotted. If samples were grouped together, ancestry values were plotted at the centroid of the group. (**C, D, G, H**) Admixture barplots. Each bar represents one individual. Samples are grouped based on their assignment to a genetic cluster (based on *K*=9): M: Mideast, E: East, W: West, S: South, O: other. (A) Historical North America. (B) Historical Europe. (C) Historical North America. (D) Historical Europe. (E) Modern North America. (F) Modern Europe. (G) Modern North America. (H) Modern Europe.

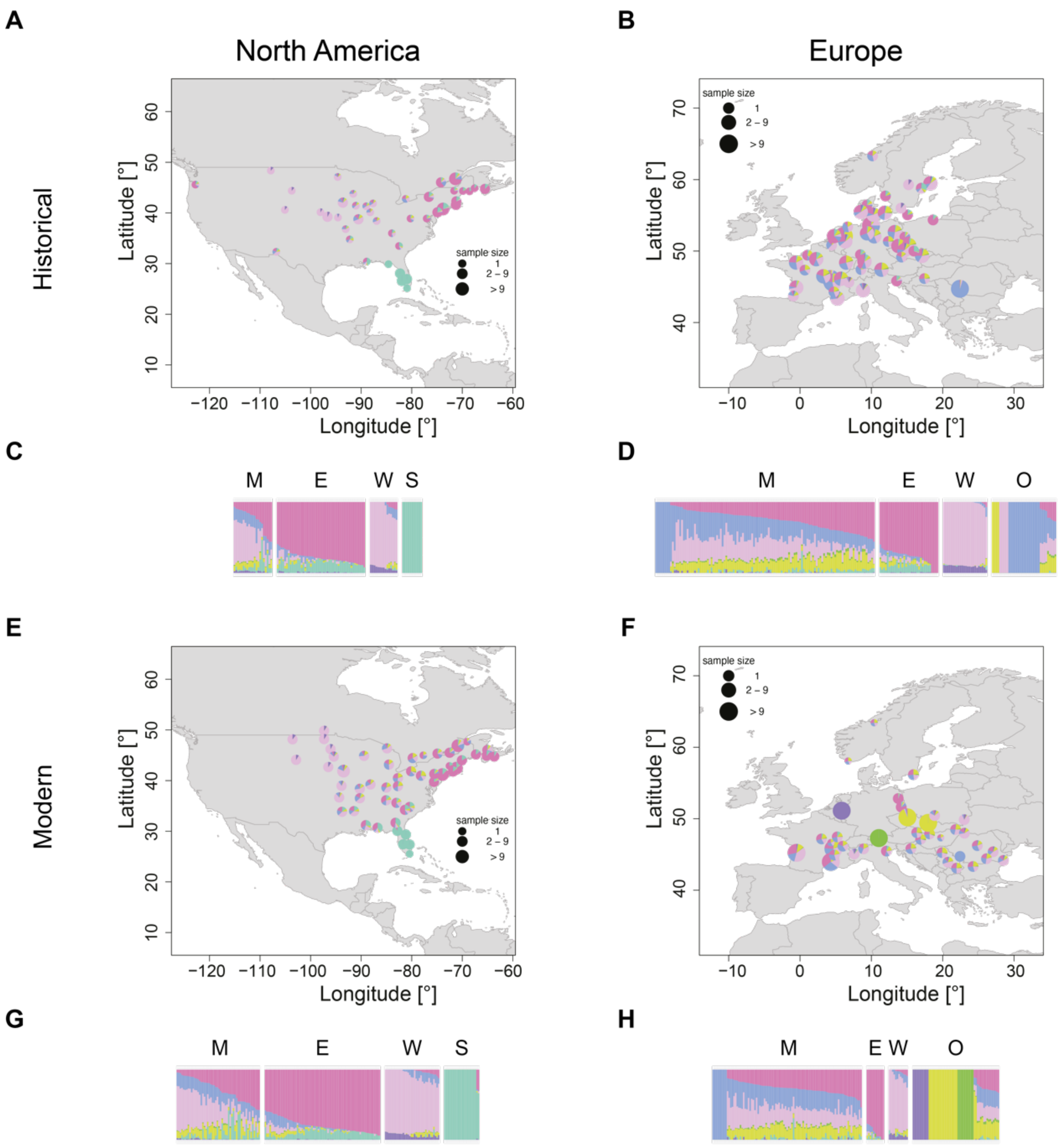

Fig. S15.

Population structure obtained from NGSadmix for K=7. The NGSadmix run of a joint analysis including all samples with the highest likelihood was used for plotting. The same color scheme was used across all panels. (**A, B, E, F**) Admixture maps. Samples within 100 km were grouped together and the average ancestry across those groups was plotted. If samples were grouped together, ancestry values were plotted at the centroid of the group. (**C, D, G, H**) Admixture barplots. Each bar represents one individual. Samples are grouped based on their assignment to a genetic cluster (based on K=9): M: Mideast, E: East, W: West, S: South, O: other. (A) Historical North America. (B) Historical Europe. (C) Historical North America. (D) Historical Europe. (E) Modern North America. (F) Modern Europe. (G) Modern North America. (H) Modern Europe.

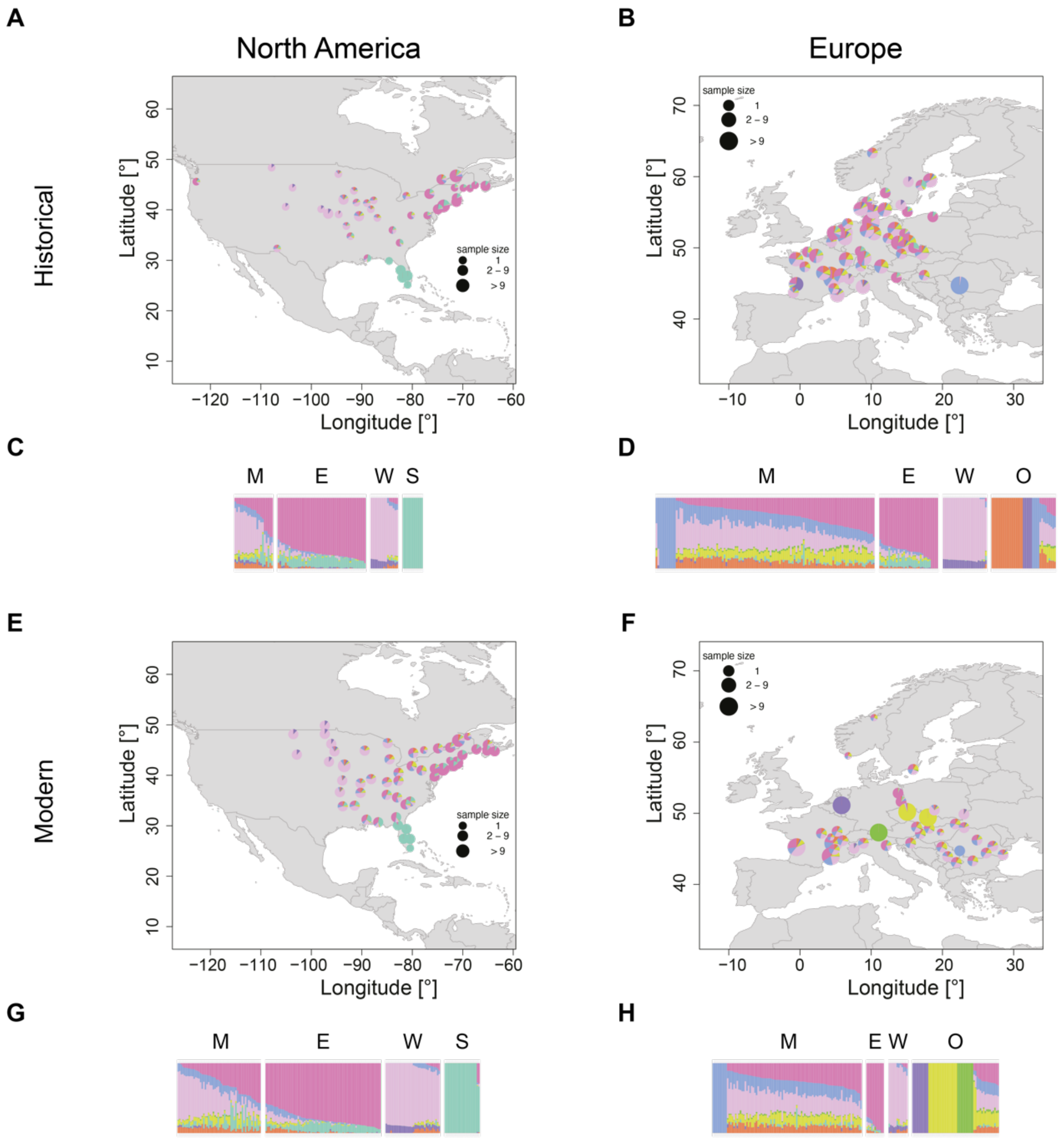

Fig. S16.

Population structure obtained from NGSadmix for K=8. The NGSadmix run of a joint analysis including all samples with the highest likelihood was used for plotting. The same color scheme was used across all panels. (**A, B, E, F**) Admixture maps. Samples within 100 km were grouped together and the average ancestry across those groups was plotted. If samples were grouped together, ancestry values were plotted at the centroid of the group. (**C, D, G, H**) Admixture barplots. Each bar represents one individual. Samples are grouped based on their assignment to a genetic cluster (based on *K*=9): M: Mideast, E: East, W: West, S: South, O: other. (A) Historical North America. (B) Historical Europe. (C) Historical North America. (D) Historical Europe. (E) Modern North America. (F) Modern Europe. (G) Modern North America. (H) Modern Europe.

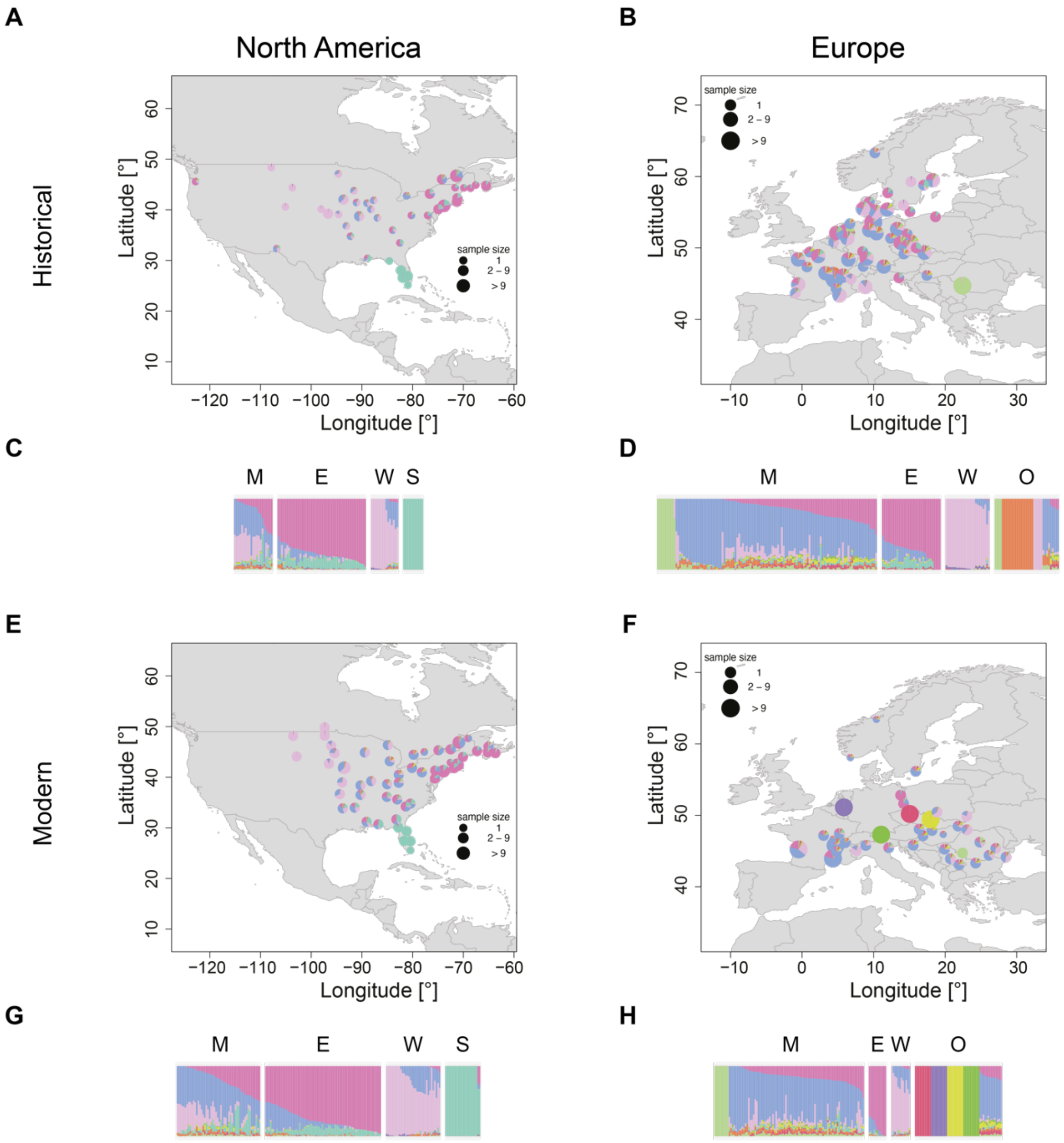

Fig. S17.

Population structure obtained from NGSadmix for K=10. The NGSadmix run of a joint analysis including all samples with the highest likelihood was used for plotting. The same color scheme was used across all panels. (**A, B, E, F**) Admixture maps. Samples within 100 km were grouped together and the average ancestry across those groups was plotted. If samples were grouped together, ancestry values were plotted at the centroid of the group. (**C, D, G, H**) Admixture barplots. Each bar represents one individual. Samples are grouped based on their assignment to a genetic cluster (based on *K*=9): M: Mideast, E: East, W: West, S: South, O: other. (A) Historical North America. (B) Historical Europe. (C) Historical North America. (D) Historical Europe. (E) Modern North America. (F) Modern Europe. (G) Modern North America. (H) Modern Europe.

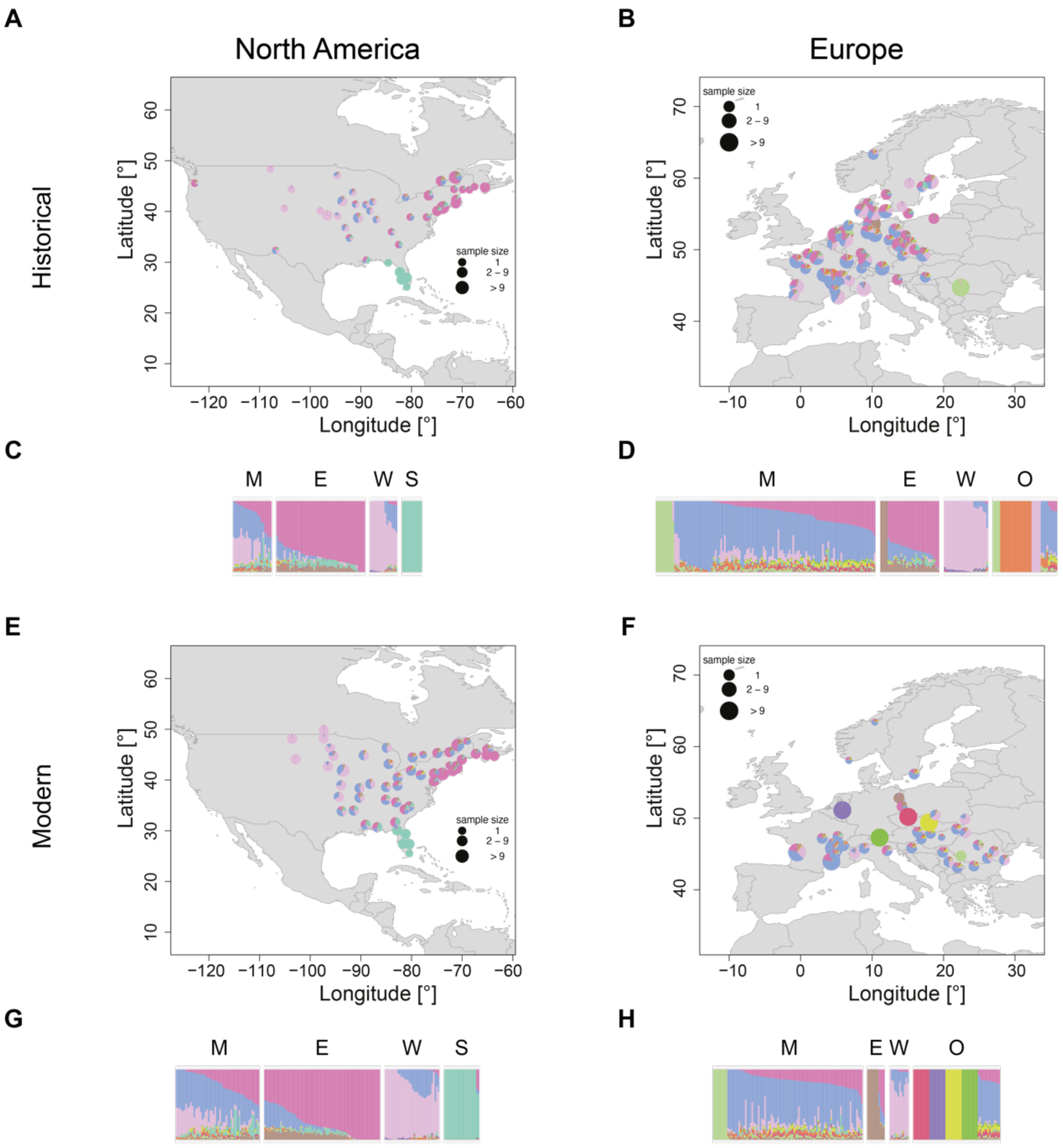

Fig. S18.

Population structure obtained from NGSadmix for K=11. The NGSadmix run of a joint analysis including all samples with the highest likelihood was used for plotting. The same color scheme was used across all panels. (**A, B, E, F**) Admixture maps. Samples within 100 km were grouped together and the average ancestry across those groups was plotted. If samples were grouped together, ancestry values were plotted at the centroid of the group. (**C, D, G, H**) Admixture barplots. Each bar represents one individual. Samples are grouped based on their assignment to a genetic cluster (based on *K*=9): M: Mideast, E: East, W: West, S: South, O: other. (A) Historical North America. (B) Historical Europe. (C) Historical North America. (D) Historical Europe. (E) Modern North America. (F) Modern Europe. (G) Modern North America. (H) Modern Europe.

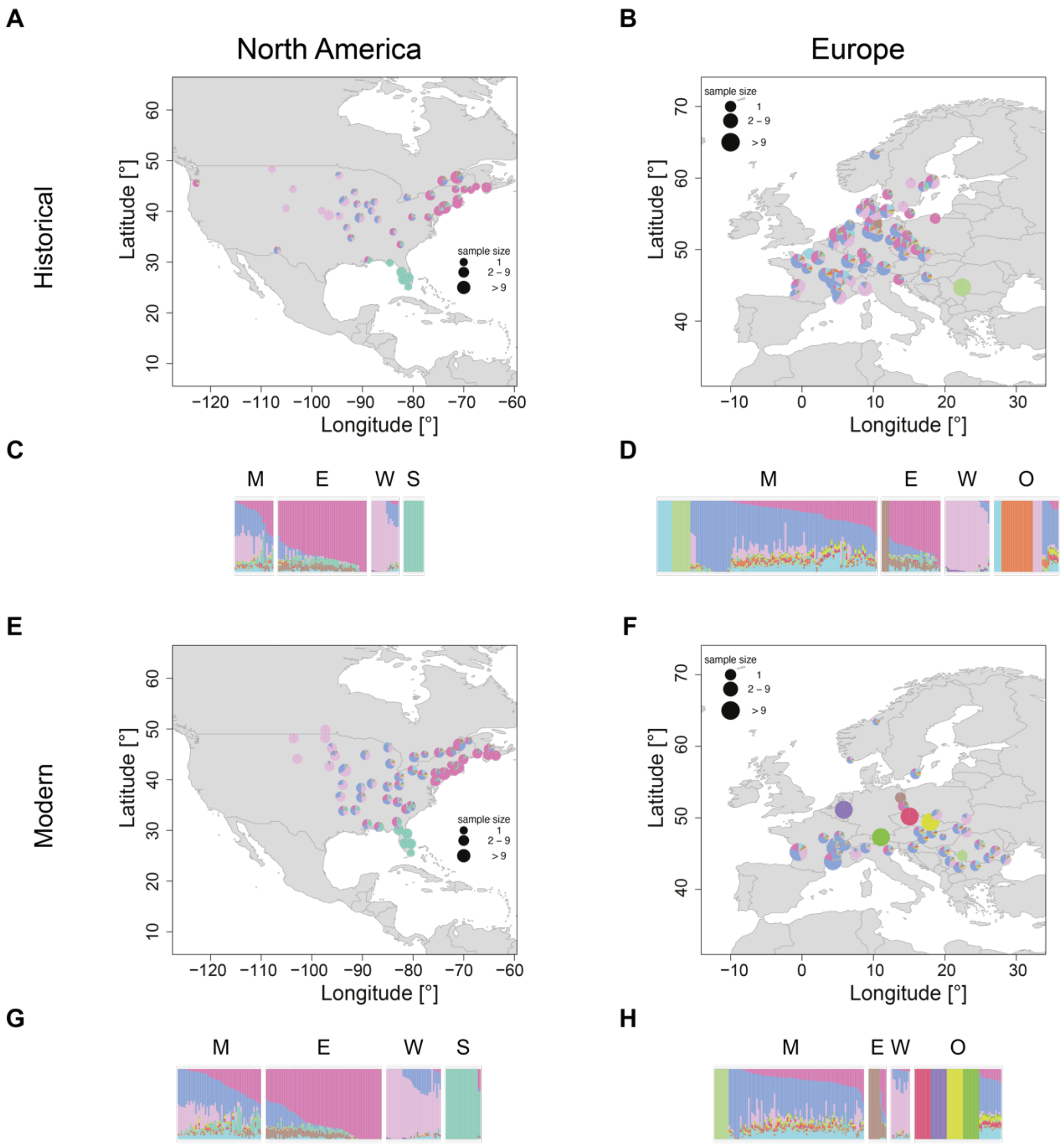

Fig. S19.

Population structure obtained from NGSadmix for K=12. The NGSadmix run of a joint analysis including all samples with the highest likelihood was used for plotting. The same color scheme was used across all panels. (**A, B, E, F**) Admixture maps. Samples within 100 km were grouped together and the average ancestry across those groups was plotted. If samples were grouped together, ancestry values were plotted at the centroid of the group. (**C, D, G, H**) Admixture barplots. Each bar represents one individual. Samples are grouped based on their assignment to a genetic cluster (based on *K*=9): M: Mideast, E: East, W: West, S: South, O: other. (A) Historical North America. (B) Historical Europe. (C) Historical North America. (D) Historical Europe. (E) Modern North America. (F) Modern Europe. (G) Modern North America. (H) Modern Europe.

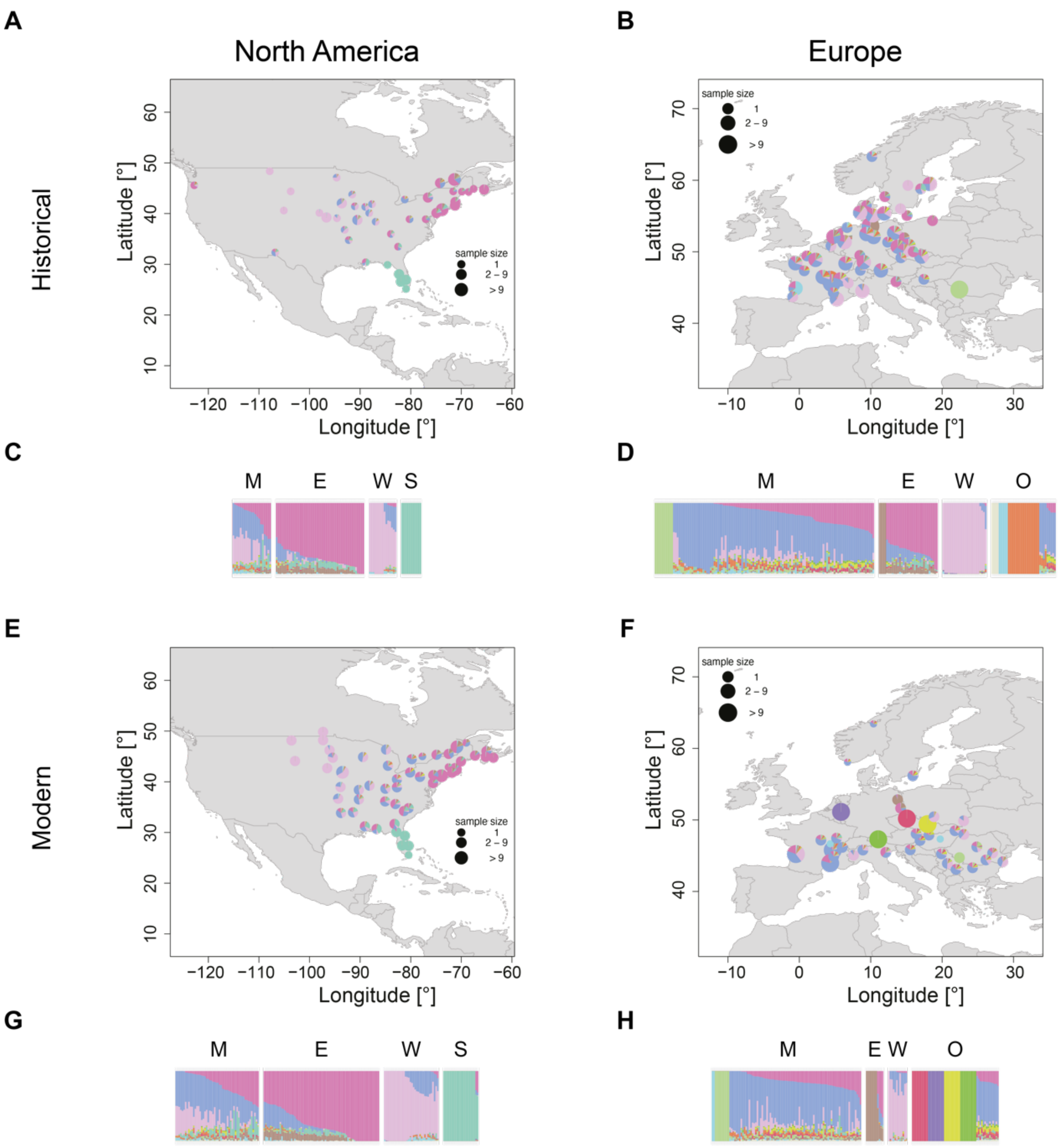

Fig. S20.

Population structure obtained from NGSadmix for K=13. The NGSadmix run of a joint analysis including all samples with the highest likelihood was used for plotting. The same color scheme was used across all panels. (**A, B, E, F**) Admixture maps. Samples within 100 km were grouped together and the average ancestry across those groups was plotted. If samples were grouped together, ancestry values were plotted at the centroid of the group. (**C, D, G, H**) Admixture barplots. Each bar represents one individual. Samples are grouped based on their assignment to a genetic cluster (based on K=9): M: Mideast, E: East, W: West, S: South, O: other. (A) Historical North America. (B) Historical Europe. (C) Historical North America. (D) Historical Europe. (E) Modern North America. (F) Modern Europe. (G) Modern North America. (H) Modern Europe.

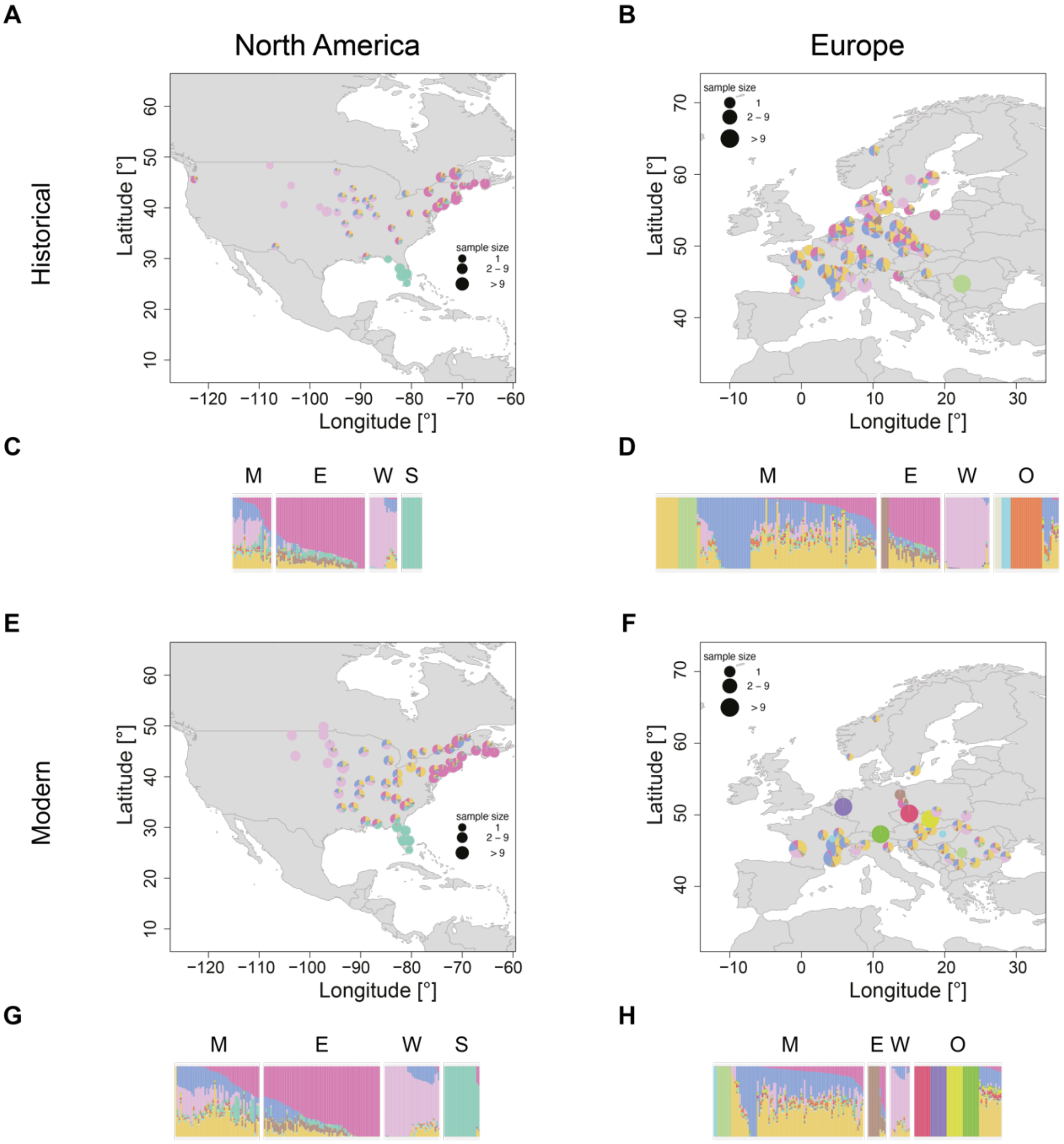

Fig. S21.

Population structure obtained from NGSadmix for K=14. The NGSadmix run of a joint analysis including all samples with the highest likelihood was used for plotting. The same color scheme was used across all panels. (**A, B, E, F**) Admixture maps. Samples within 100 km were grouped together and the average ancestry across those groups was plotted. If samples were grouped together, ancestry values were plotted at the centroid of the group. (**C, D, G, H**) Admixture barplots. Each bar represents one individual. Samples are grouped based on their assignment to a genetic cluster (based on *K*=9): M: Mideast, E: East, W: West, S: South, O: other. (A) Historical North America. (B) Historical Europe. (C) Historical North America. (D) Historical Europe. (E) Modern North America. (F) Modern Europe. (G) Modern North America. (H) Modern Europe.

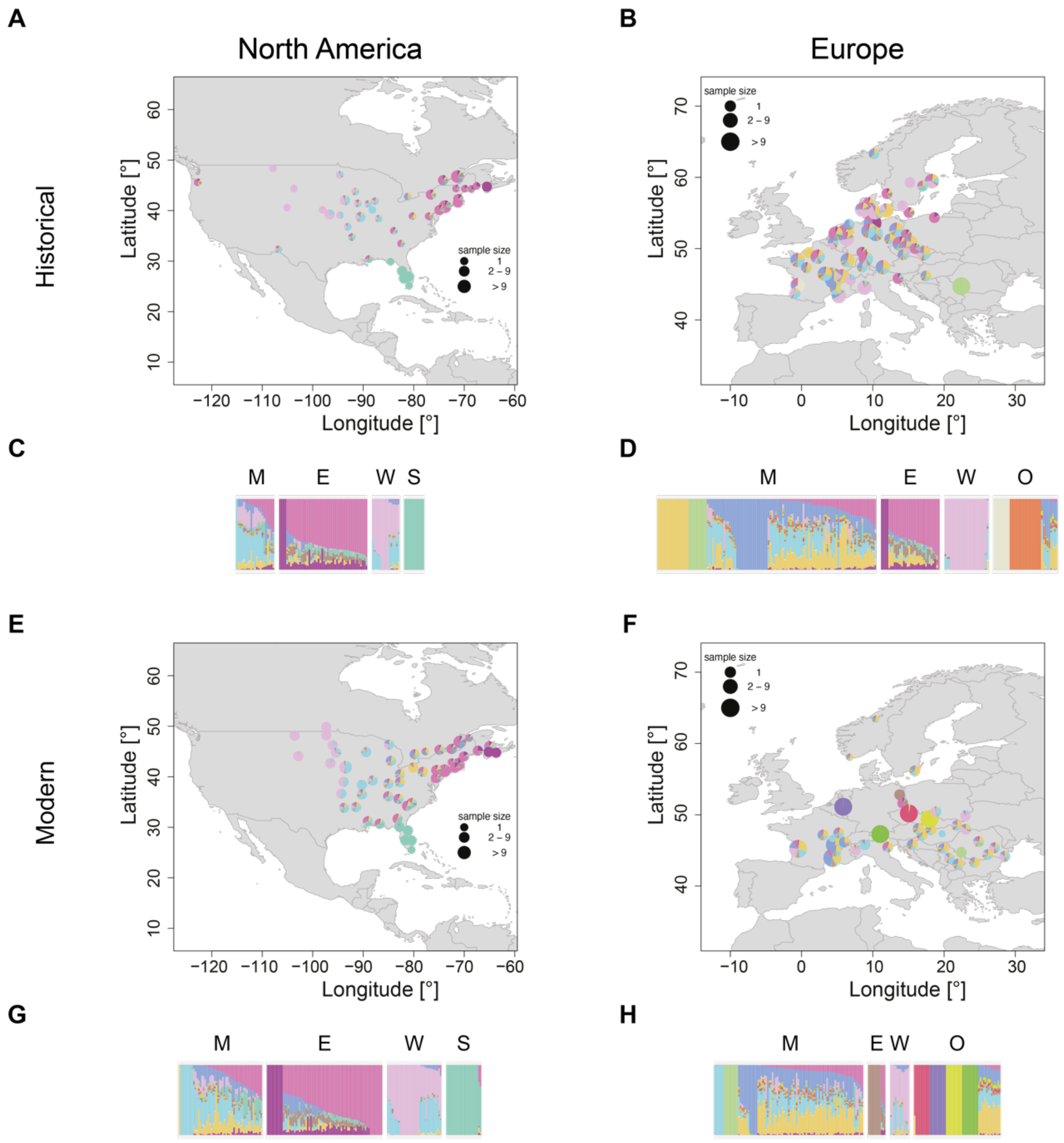

Fig. S22.

Population structure obtained from NGSadmix for K=15. The NGSadmix run of a joint analysis including all samples with the highest likelihood was used for plotting. The same color scheme was used across all panels. (**A, B, E, F**) Admixture maps. Samples within 100 km were grouped together and the average ancestry across those groups was plotted. If samples were grouped together, ancestry values were plotted at the centroid of the group. (**C, D, G, H**) Admixture barplots. Each bar represents one individual. Samples are grouped based on their assignment to a genetic cluster (based on K=9): M: Mideast, E: East, W: West, S: South, O: other. (A) Historical North America. (B) Historical Europe. (C) Historical North America. (D) Historical Europe. (E) Modern North America. (F) Modern Europe. (G) Modern North America. (H) Modern Europe.

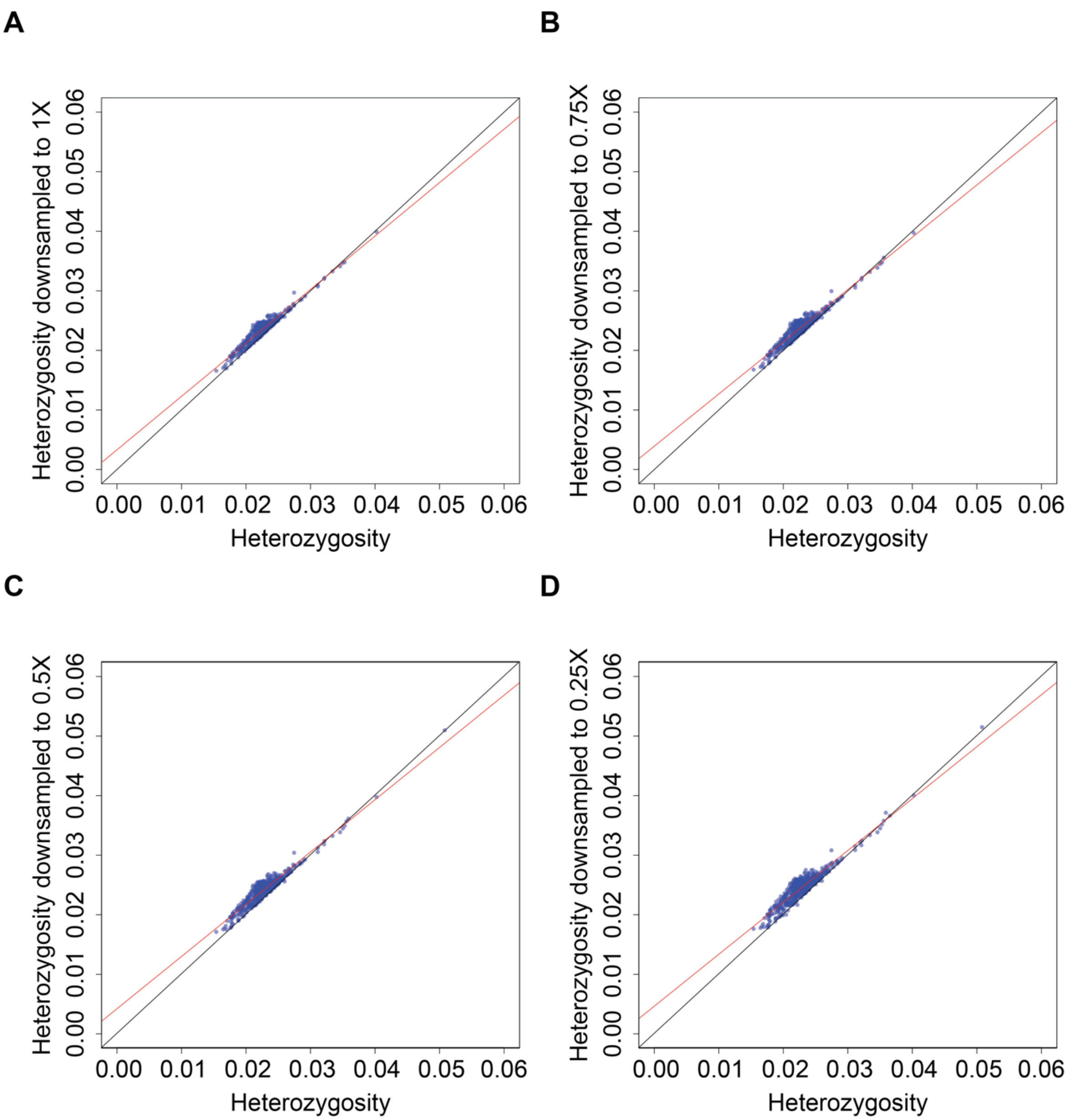

Fig. S23.

Correlation of heterozygosity using all reads per sample vs. downsampled to (**A**) 1X sequencing depth (Adjusted R-squared: 0.9255, p-value: < 2.2e-16), (**B**) 0.75X sequencing depth (Adjusted R-squared: 0.9101, p-value: < 2.2e-16), (**C**) 0.5X sequencing depth (Adjusted R-squared: 0.9046, p-value: < 2.2e-16), (**D**) 0.25X sequencing depth (Adjusted R-squared: 0.8828, p-value: < 2.2e-16). The black line represents a perfect correlation and the red line represents the linear regression line.

Table S4.

Population-level statistics. The estimate of Tajima's D is based on the mean value across all windows in the sliding window analysis. Heterozygosity was estimated for each sample individually and the mean across samples in a population is reported here. Effective population size was estimated based on the nucleotide diversity, assuming a generation time of one year and a mutation rate of 1 x 10^{-8} substitutions/site/generation.

**Population**	**Sample size**	**Tajima's D**	**Heterozygosity**	**Nucleotide diversity**	**Effective population size (N_e)**
Modern West	34	-1.396072	0.02332	0.0272	1,138,551
Historical West	15	-1.488506	0.02478	0.0253	1,048,179
Modern Mideast	50	-1.434849	0.02302	0.0273	1,197,444
Historical Mideast	20	-1.571675	0.02903	0.0260	1,155,252
Modern South	22	-1.037859	0.02293	0.0247	857,481
Historical South	11	-1.133698	0.02337	0.0225	782,524
Modern East	71	-1.260844	0.02237	0.0265	1,083,989
Historical East	46	-1.679911	0.02447	0.0240	1,236,892
Modern Europe	170	-1.394707	0.02181	0.0268	1,243,678
Historical Europe	211	-1.904119	0.02273	0.0252	1,788,097

Table S1. (separate file)

Overview of herbarium and contemporary *Ambrosia artemisiifolia* samples and sample preparation information used in this study. Abbreviations of source herbaria: B: Botanischer Garten und Botanosches Museum Berlin, Germany; BR: Meise Botanic Garden, Belgium; BRNU: Masaryk University, Czech Republic; C: University of Copenhagen, Denmark; FI: Natural History Museum Firenze, Italy; G: Conservatoire et Jardin botaniques de la Ville de Genève, Switzerland; GH: Harward University, USA; GOET: Universität Göttingen, Germany; GZU: Karl-Franzens-Universität Graz, Austria; HBG: University of Hamburg, Germany; I_ Alexander Ioan Cuza University, Romania; University of Agricultural Sciences and Veterinary Medicine "Ion Ionescu de la Brad", Romania; JE: Friedrich Schiller Universität Jena, Germany; L: Naturalis Leiden, Netherlands; LD: Lund University, Sweden; LY: Université Claude Bernard Lyon, France; MARS: Aix-Marseille Université, France; MO: Missouri Botanical Garden, USA; MPU: Université de Montpellier, France; NEBC: New England Botanical Club, USA; NEU: Université de Neuchâtel, Switzerland; NY: New York Botanical Garden, USA; P: Muséum National d'Histoire Naturelle, France; PH: Academy of Natural Science, USA; PR: National Museum Prague, Czech Republic; PRA: Insitute of Botany, Academy of Sciences, Pruhonice, Czech Republic; PRC: Charles University Prague, Czech Republic; QFA: Université Laval Québec, Canada; ROZ: Sterdoceské Muzeum Roztoky, Czech Republic; S: Swedish Museum of Natural History, Sweden; STU: Staatliches Museum für Naturkunde Stuttgard, Germany; TRH: Norwegian University of Science and Technology, Norway; UPS: Museum of Evolution Lund, Sweden; US: Smithonian Institution, USA; W: Naturhistorisches Museum Wien, Austria; W: Universität Wien, Austria. For samples grown from seeds, location and collection date represent that of the seeds.

Table S2. (separate file)

Ancient DNA damage statistics for Ambrosia artemisiifolia herbarium specimens. The C to T base misincorporation at the first base is given for each library of each sample. The endogenous content is estimated as the fraction of reads mapping against the A. artemisiifolia refernece genome before filtering for PCR duplicates and mapping quality.

Table S3. (separate file)

Spatial groups statistics. The estimate of Tajima's D is based on the mean value across all windows in the sliding window analysis. Heterozygosity was estimated for each sample individually and the mean across samples in a spatial group is reported here. Effective population size was estimated based on the nucleotide diversity, assuming a generation time of one year and a mutation rate of 1 x 10-8 substitutions/site/generation.

Table S5. (separate file)

Significantly enriched GO terms in Fst-outlier windows.

Table S6. (separate file)

Top outlier SNPs (Z>50) located in gene regions.

Table S7. (separate file)

Prevalence of plant pathogens in the different ranges and time periods. Ranges that have a significantly higher prevalence (Welch two sample t-test p-value < 0.05) within a time period are marked in yellow.

Table S8. (separate file)

Overview of outgroup samples from different *Ambrosia* species used to identify putative hybrids and misidentifications in the *Ambrosia artemisiifolia* dataset and their sample preparation methods. For all samples, leaf material from herbarium specimens were used for DNA extraction. Abbreviations of source herbaria: A: Harvard University, USA; GH: Gray Herbarium, Harvard University herbaria, USA; H: Finnish Museum of Natural History, Finland; MASS: University of Massachusetts, USA; NYB: New York Botanical Garden, USA; P: Muséum National d'Histoire Naturelle Paris, France; RBGE: Royal Botanical Garden Edinburgh, UK; S: Swedish Museum of Natural History, Sweden; UC: University Herbarium, University of California, Berkeley, USA.

Table S9. (separate file)

F_{ST} between spatial groups. The weighted estimate of F_{ST} between spatial group 1 and spatial group 2 are given.
